# SARS-CoV-2 N Protein Antagonizes Stress Granule Assembly and IFN Production by Interacting with G3BPs to Facilitate Viral Replication

**DOI:** 10.1128/jvi.00412-22

**Published:** 2022-06-02

**Authors:** Hainan Liu, Yu Bai, Xun Zhang, Ting Gao, Yue Liu, Entao Li, Xuefeng Wang, Zheng Cao, Lin Zhu, Qincai Dong, Yong Hu, Guangfei Wang, Caiwei Song, Xiayang Niu, Tong Zheng, Di Wang, Zijing Liu, Yanwen Jin, Ping Li, Xiuwu Bian, Cheng Cao, Xuan Liu

**Affiliations:** a Beijing Institute of Biotechnology, Beijing, China; b Anhui University, Hefei, China; c Hubei University of Technology, Wuhan, China; d Changchun Veterinary Research Institute, Chinese Academy of Agricultural Sciences, Changchun, Jilin, China; e First Affiliated Hospital, Army Medical University, Chongqing, China; The Peter Doherty Institute for Infection and Immunity

**Keywords:** G3BP1, nucleocapsid protein, SARS-CoV-2, stress granule

## Abstract

SARS-CoV-2 is the causative agent of the ongoing pandemic of coronavirus disease 2019 (COVID-19) and poses a significant threat to global health. N protein (NP), which is a major pathogenic protein among betacoronaviruses, binds to the viral RNA genome to allow viral genome packaging and viral particle release. Recent studies showed that NP antagonizes interferon (IFN) induction and mediates phase separation. Using live SARS-CoV-2 viruses, this study provides solid evidence showing that SARS-CoV-2 NP associates with G3BP1 and G3BP2 *in vitro* and *in vivo*. NP^SARS-CoV-2^ could efficiently suppress G3BP-mediated SG formation and potentiate viral infection by overcoming G3BP1-mediated antiviral innate immunity. G3BP1 conditional knockout mice (*g3bp1^fl/fL^*, *Sftpc*-Cre) exhibit significantly higher lung viral loads after SARS-CoV-2 infection than wild-type mice. Our findings contribute to the growing body of knowledge regarding the pathogenicity of NP^SARS-CoV-2^ and provide insight into new therapeutics targeting NP^SARS-CoV-2^.

**IMPORTANCE** In this study, by *in vitro* assay and live SARS-CoV-2 virus infection, we provide solid evidence that the SARS-CoV-2 NP associates with G3BP1 and G3BP2 *in vitro* and *in vivo*. NP^SARS-CoV-2^ could efficiently suppress G3BP-mediated SG formation and potentiate viral infection by overcoming antiviral innate immunity mediated by G3BP1 in A549 cell lines and G3BP1 conditional knockout mice (*g3bp1*-cKO) mice, which provide in-depth evidence showing the mechanism underlying NP-related SARS-CoV-2 pathogenesis through G3BPs.

## INTRODUCTION

Coronavirus disease 2019 (COVID-19), caused by severe acute respiratory syndrome coronavirus 2 (SARS‐CoV‐2), poses a significant threat to global health. Similar to other betacoronaviruses, such as SARS-CoV and Middle East respiratory syndrome coronavirus (MERS-CoV), SARS-CoV-2 is an enveloped, single-stranded, positive-sense RNA virus with a 30-kb genome that encodes four structural proteins, spike (S), envelope (E), membrane (M), and nucleocapsid (N). N protein (NP) binds to the viral RNA genome to allow viral genome packaging and viral particle release (1, 2). Similar to NPs of SARS-CoV and MERS-CoV, NP of SARS-CoV-2 suppresses type I interferon (IFN) induction by targeting RIG-I signaling (3–6), but the molecular details that underlie this process are poorly understood.

Stress granules (SGs) are dynamic large cytoplasmic aggregates composed of mRNAs and proteins that assemble to respond to cellular stresses, such as oxidative stress and viral infection. SG formation results in the recruitment of virus sensors and robust translation arrest, which can block viral gene expression (7). G3BP1 and G3BP2, which associate with mRNA, are vital components for SG nucleation. Moreover, G3BP1 inhibits viral replication by positively regulating RIG-I- and cGAS-mediated cellular antiviral responses (8, 9). The capsid proteins of several positive-sense single-stranded RNA viruses, such as Zika virus (ZIKV), yellow fever virus (YFV), and Murray Valley encephalitis virus (MVEV), reportedly hijack host SG proteins to antagonize SG formation and modulate host stress responses (10), which suggests that SG-mediated innate immunity plays important roles against viral infection. Recently, SARS-CoV-2 NP was predicted to be associated with G3BP1 and G3BP2 by affinity purification-mass spectrometry (MS) analysis (11), and their interaction was recently demonstrated to impair SG formation to promote viral replication, as demonstrated by immunofluorescence, pseudovirus infection, and SARS-CoV-2 infection (12–16). However, more information about the NP-G3BPs interactions and more data on infected tissues from animals and even patients with COVID-19 need to be further assessed. Whether the NPs of other highly pathogenic coronaviruses are involved in the regulation of G3BPs-involved host innate immunity is also worth further investigation.

In this study, the binding of betacoronavirus NPs with host G3BP1 and G3BP2 was systematically investigated *in vivo* and *in vitro*. SG assembly was significantly reduced by NP expression and by SARS-CoV-2 infection. NP^SARS-CoV-2^ inhibited G3BP1-related, RIG-I-mediated interferon (IFN) production to facilitate viral replication. Furthermore, *g3bp1* conditional knockout mice exhibit significantly higher lung viral loads after SARS-CoV-2 infection than wild-type mice. Our findings contribute to the growing body of knowledge regarding the pathogenicity of SARS-CoV-2 NP and provide insight into new therapeutics targeting SARS-CoV-2 NP.

## RESULTS

### G3BPs interact directly with NPs of betacoronaviruses.

**FIG 1:**
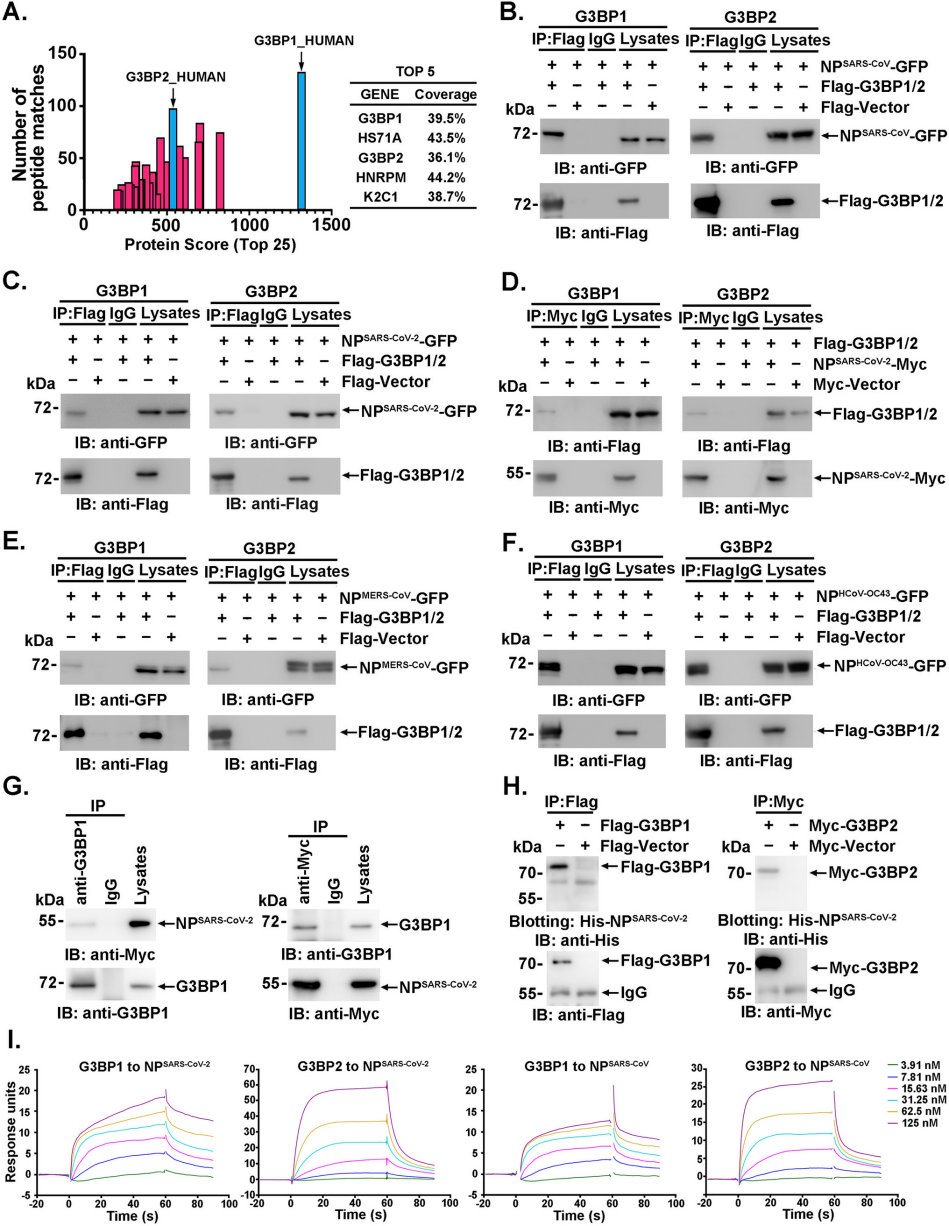
G3BPs interact directly with NPs of betacoronaviruses. (A) Lysates of 293T cells transfected with Flag-tagged NP^SARS-CoV^ were immunoprecipitated with anti-Flag antibody and then subjected to LC-MS/MS analysis to identify the interacting proteins. The protein scores, exclusive spectrum count, and detected protein coverage were analyzed using MaxQuant software. (B to G) Lysates of 293T cells expressing the indicated plasmids were subjected to immunoprecipitation and immunoblot analysis with the indicated antibodies. (H) Lysates of 293T cells transfected with the indicated plasmids were subjected to SDS-PAGE and blotted onto a PVDF membrane. The PVDF membrane was incubated with purified recombinant His-tagged NP^SARS-CoV-2^ and then detected with anti-His antibody. (I) SPR assays were performed with the indicated purified proteins to detect the direct interactions between G3BP1/G3BP2 and NP^SARS-CoV-2^. *K_D_* values were calculated using software.

To identify proteins associated with SARS-CoV NP, we previously expressed Flag-tagged SARS-CoV NP (NP^SARS-CoV^) in 293 cells and immunoprecipitated the cell lysates with anti-Flag antibody (3). The proteins that coprecipitated with NPs were identified by liquid chromatography-tandem mass spectrometry (LC-MS/MS) analysis after SDS-PAGE. G3BP1 and G3BP2 were both confidently observed in the SARS-CoV NP immunoprecipitates due to the high exclusive spectrum counts, high protein scores and high coverage (Fig. 1A; see Table S1 in the supplemental material). These results suggested that SARS-CoV NP may interact with G3BPs. To investigate this interaction, lysates of 293T cells cotransfected with NP^SARS-CoV^-green fluorescent protein (GFP) and Flag-G3BP1 were subjected to anti-Flag immunoprecipitation followed by immunoblotting with anti-GFP antibody. NP^SARS-CoV^-GFP coprecipitated with Flag-G3BP1 but not the Flag tag. As a control, no NP^SARS-CoV^-GFP was observed in the IgG immunoprecipitates. These results suggested that NP^SARS-CoV^ may associate with G3BP1 and G3BP2 (Fig. 1B). Next, the associations between G3BPs and NPs of other betacoronaviruses, such as SARS-CoV-2, MERS-CoV, and human coronavirus (HCoV)-OC43, were investigated. The presence of each NP of coronavirus was detected in the anti-Flag or anti-Myc immunoprecipitates of 293T cells ectopically expressing NP and Flag-G3BP1 or Flag-G3BP2 (Fig. 1C to F). Furthermore, endogenous G3BP1 bound to ectopically expressed NP^SARS-CoV-2^ in 293T cells (Fig. 1G, left). Similar results were also obtained in reciprocal experiments (Fig. 1G, right). These results collectively demonstrate a broad-spectrum interaction between G3BPs and NPs of betacoronaviruses.

Because COVID-19 remains a worldwide pandemic, we focused on the interaction of G3BPs with SARS-CoV-2 NP. The direct interaction was then validated by far-Western blot and surface plasmon resonance (SPR) analyses. Anti-Flag immunoprecipitates prepared from 293T cells expressing Flag-G3BP1 or Flag-vector were subjected to SDS-PAGE. The proteins were transferred to a polyvinylidene difluoride (PVDF) membrane and then blotted with soluble His-NP^SARS-CoV-2^, and the results showed that His-NP^SARS-CoV-2^ directly bound G3BP1, but not IgG, in the same lane *in vitro* (Fig. 1H, left). Consistently, G3BP2 was also observed to interact directly with NP^SARS-CoV-2^ (Fig. 1H, right). The kinetics of the interaction was further assessed by SPR analysis, and NP^SARS-CoV-2^ was found to bind with G3BP1 or G3BP2 with equilibrium dissociation constant (*K_D_*) values of 2.69 × 10^−8^ M and 1.03 × 10^−7^ M, respectively (Fig. 1I, two left panels). High binding affinity was also observed between NP^SARS-CoV^ and G3BPs (*K_D_* values, 2.57 × 10^−8^ M and 3.85 × 10^−8^ M; Fig. 1I, two right panels). These results supported the assertion that both G3BP1 and G3BP2 interacted strongly and directly with NP^SARS-CoV-2^ (with a *K_D_* of 10^−8^ to 10^−7^ M). Notably, NP^SARS-CoV-2^ showed a stronger (~4-fold) interaction with G3BP1 than G3BP2.

**FIG 2:**
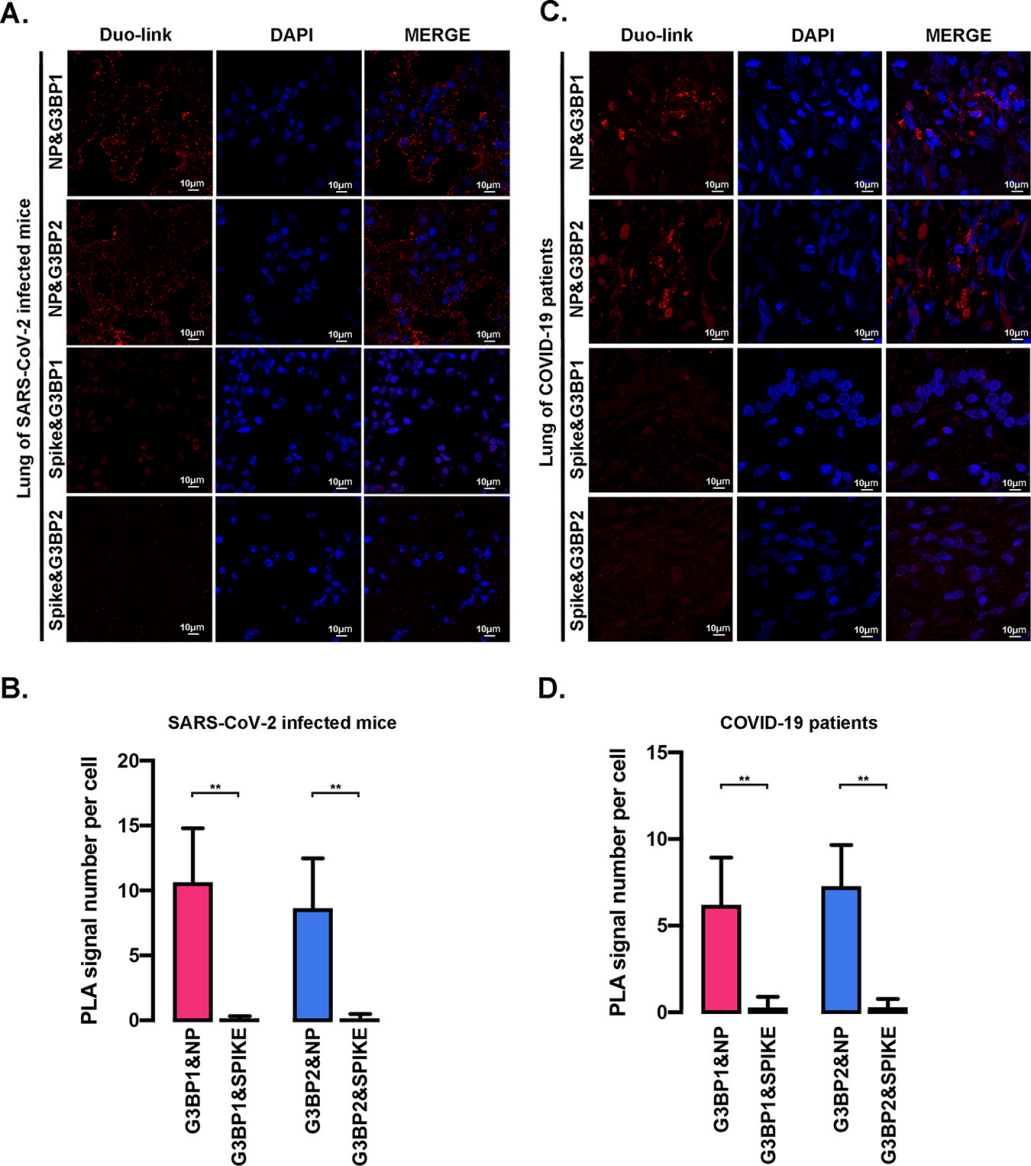
Analysis of interactions between G3BPs and NP^SARS-CoV-2^ using live SARS-CoV-2 virus. (A and C) *In situ* PLAs were performed using the indicated antibodies to evaluate lung sections from mice (A) and patients (C). The Duolink signals were subsequently visualized in red, and the nuclei were stained with DAPI (4′,6-diamidino-2-phenylindole; blue). (B and D) The relative fluorescence intensity per cell shown in panels A and C was analyzed using ImageJ software (at least 20 cells). All quantitative data are shown as the means ± SDs. **, *P* < 0.05.

To demonstrate that NP^SARS-COV-2^ interacts with G3BPs in SARS-CoV-2 infection, the *in situ* interaction was subjected in the lungs of mice infected with mouse-adapted SARS-CoV-2 (12) through a Duolink proximity ligation assay (PLA) with anti-NP and anti-G3BP1 (or G3BP2) antibodies. In agreement with the findings reported by Zheng (13), NP^SARS-CoV-2^ is associated with G3BP1 or G3BP2 *in situ* (Fig. 2A and B). As a control, little, if any interaction was observed between the S protein of SARS-CoV-2 and G3BP1 (or G3BP2) (Fig. 2A and B). Notably, the *in situ* interaction of NP^SARS-CoV-2^ with G3BPs was observed in the lungs of patients with COVID-19 with the S protein of SARS-CoV-2 and G3BP1 (or G3BP2) as a control (Fig. 2C and D). These results demonstrated that the nucleocapsid protein interacts with G3BPs in SARS-CoV-2 infection. Collectively, these findings demonstrate that G3BPs, including G3BP1 and G3BP2, directly interact with NP^SARS-COV-2^.

### Domains involved in the G3BPs-NP^SARS-CoV-2^ association.

**FIG 3:**
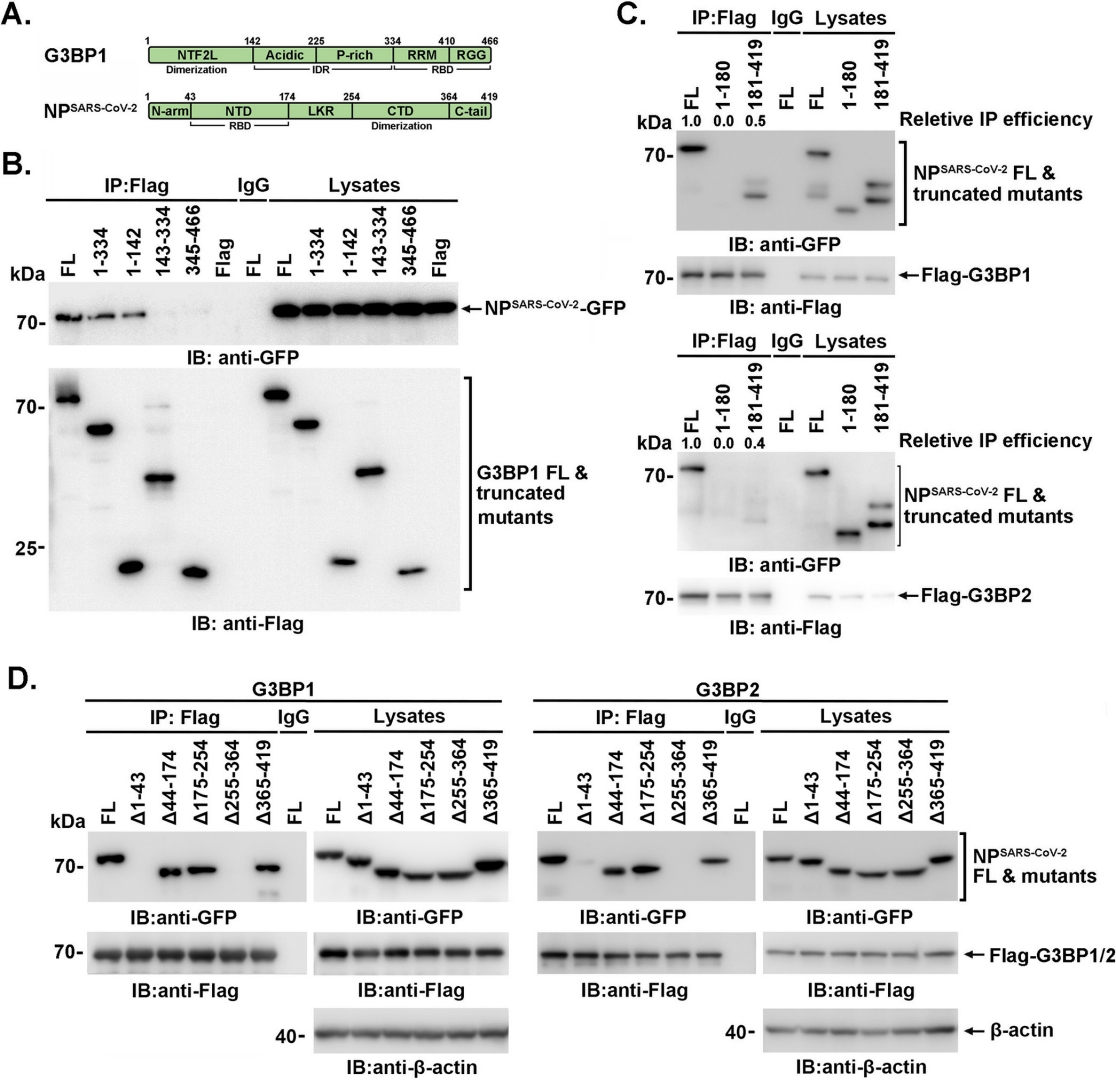
Domains involved in the G3BPs-NP^SARS-CoV-2^ association. (A) Schematic diagram of the domains of G3BP1 and SARS-CoV-2 NP. IDR, intrinsically disordered region; RBD, RNA-binding domain. (B) 293T cells were cotransfected with NP (SARS-CoV-2) and G3BP1 or truncated mutants, and immunoprecipitation and immunoblotting were then performed with the indicated antibodies. (C and D) 293T cells coexpressing G3BP1 and NP^SARS-CoV-2^ or truncated mutants were subjected to immunoprecipitation and immunoblotting with the indicated antibodies.

To delineate the domains involved in the G3BPs-NP^SARS-CoV-2^ association, anti-Flag immunoprecipitates of full-length or truncated Flag-G3BP1 were incubated with lysates of 293T cells expressing NP^SARS-CoV-2^-GFP (Fig. 3A and B). An analysis of the adsorbates by immunoblotting with anti-GFP antibody demonstrated that the N terminus (amino acids [aa] 1 to 142) of G3BP1, termed the nuclear transport factor 2-like (NTF2L) domain, was responsible for the association with NP^SARS-CoV-2^ (Fig. 3B). Flag-G3BP1 (or Flag-G3BP2) was then cotransfected with GFP-tagged full-length or truncated NP^SARS-CoV-2^ into 293T cells. The NP^SARS-CoV-2^ (aa 181 to 419), but not NP^SARS-CoV-2^ (aa 1 to 180), was observed to associate with G3BP1, with a significantly weakened binding compared to that of full-length NP^SARS-CoV-2^ (Fig. 3C). Further, the deletion mutant of the NP confirmed that its 255-364 aa motif was indispensable for G3BP1 association (Fig. 3D). However, the 1-43 aa motif (N-terminal intrinsically disordered region [IDR], N-terminal arginine-rich motif [N-ARM]) was also observed contributing to the association.

**FIG 4:**
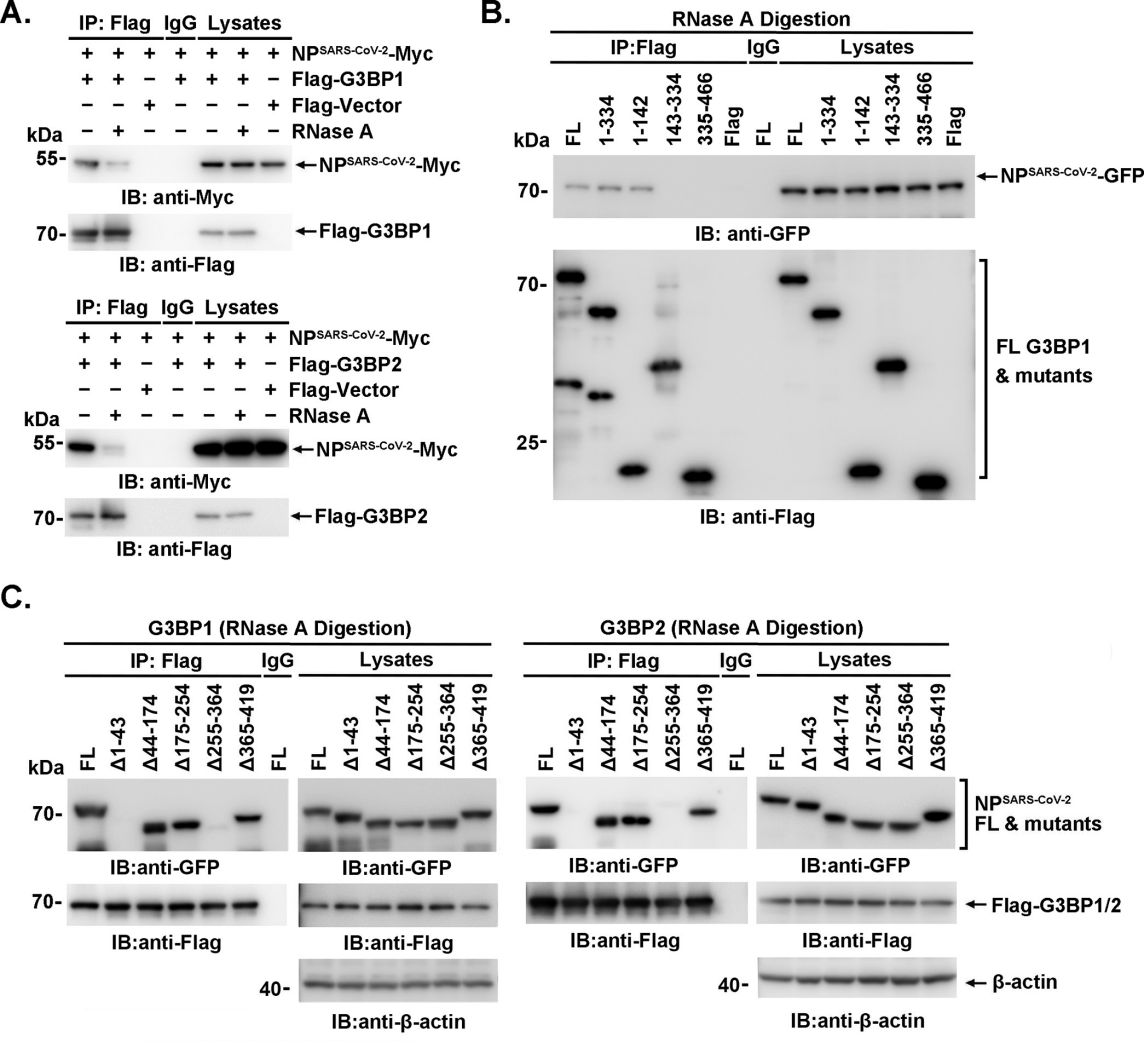
The binding of NP^SARS-CoV-2^ to G3BPs is partially dependent on the presence of RNA. (A to C) 293T cells were transfected with the indicated plasmids. After RNase A (100 μg/mL) digestion for 30 min at 37°C, the lysates were subjected to immunoprecipitation and immunoblotting using the indicated antibodies.

Furthermore, the association between NP^SARS-CoV-2^-Myc and Flag-G3BPs was significantly attenuated by RNase A digestion (Fig. 4A). Consistently, the interactions between truncated G3BP1 and NP or between G3BP1/G3BP2 and truncated NP were not completely disrupted by RNase A digestion (Fig. 4B and C). These findings suggested that the binding of NP^SARS-CoV-2^ to G3BPs is partially dependent on the presence of RNA.

### SARS-CoV-2 NP suppresses G3BP-mediated SG formation.

**FIG 5:**
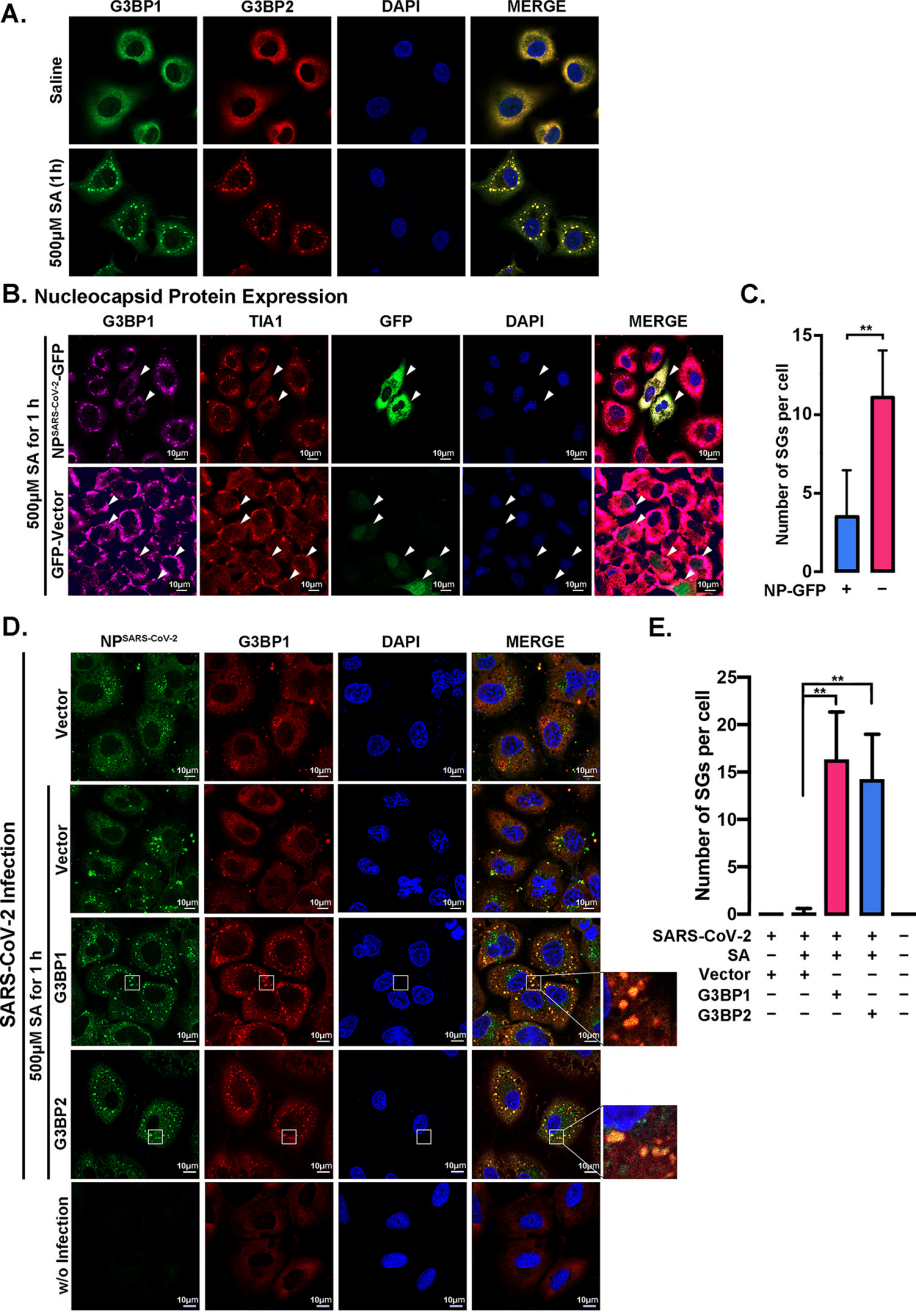
SARS-CoV-2 NP suppresses G3BP-mediated SG formation. (A) A549 cells were treated with 500 μM sodium arsenite (SA) for 1 h to induce SG assembly, and saline was used as a control. G3BP1 (green) and G3BP2 (red) were labeled to visualize SGs by immunofluorescence staining. The nuclei were stained with DAPI (blue). (B) A549 cells expressing GFP-tagged NP^SARS-CoV-2^ or GFP-vector were treated with 500 μM SA for 1 h to induce SG assembly. TIA1 (red) and G3BP1 (violet) were labeled to visualize SGs by immunofluorescence staining. The nuclei were stained with DAPI (blue). (C) The average SG number per cell (≥10) in panel B was calculated using ImageJ software. All quantitative data are shown as the means ± SDs. **, *P* < 0.05. (D) A549 cells transfected with or without a G3BP1/G3BP2 plasmid were infected with SARS-CoV-2 for 24 h and treated with or without 500 μM SA during the last hour. NP^SARS-CoV-2^ (green) and the SG marker G3BP1 (red) were detected via immunofluorescence staining. The nuclei were stained with DAPI (blue). (E) The average SG number per cell (≥10) in panel D was calculated using ImageJ software. All quantitative data are shown as the means ± SDs. **, *P* < 0.05.

NP^SARS-CoV-2^ binds to G3BPs via aa 255 to 364, and this binding event is responsible for the liquid-liquid phase separation of NP (14). Furthermore, the NTF2L domain plays vital roles in G3BP dimerization and recruitment to SGs (15). Earlier studies also suggested that NP suppresses SG formation (13, 17). Accordingly, treatment with sodium arsenite (SA) resulted in stronger colocalization between NP and G3BP1, which suggests their association, and SA-induced SG formation in A549 cells (Fig. 5A) was significantly suppressed in the cells expressing NP^SARS-CoV-2^-GFP compared with the cells not expressing NP^SARS-CoV-2^ in the same field (Fig. 5B, top, and Fig. 5C) or the cells expressing GFP only (Fig. 5B, bottom). Importantly, SA-induced SG assembly was also suppressed by SARS-CoV-2 infection in A549 cells, and SG formation could be markedly rescued by either G3BP1 or G3BP2 overexpression (Fig. 5D and E). As expected, SARS-CoV-2 NP colocalized significantly with G3BPs in SGs of infected cells. These findings demonstrate that NP^SARS-CoV-2^ suppresses G3BP-mediated SG formation, possibly by “neutralizing” the G3BPs via protein interactions because the oversupply of G3BP1 or G3BP2 could rescue the effect induced by SARS-CoV-2 infection.

### SARS-CoV-2 NP inhibits G3BP1-dependent IFN production and potentiates viral proliferation.

**FIG 6:**
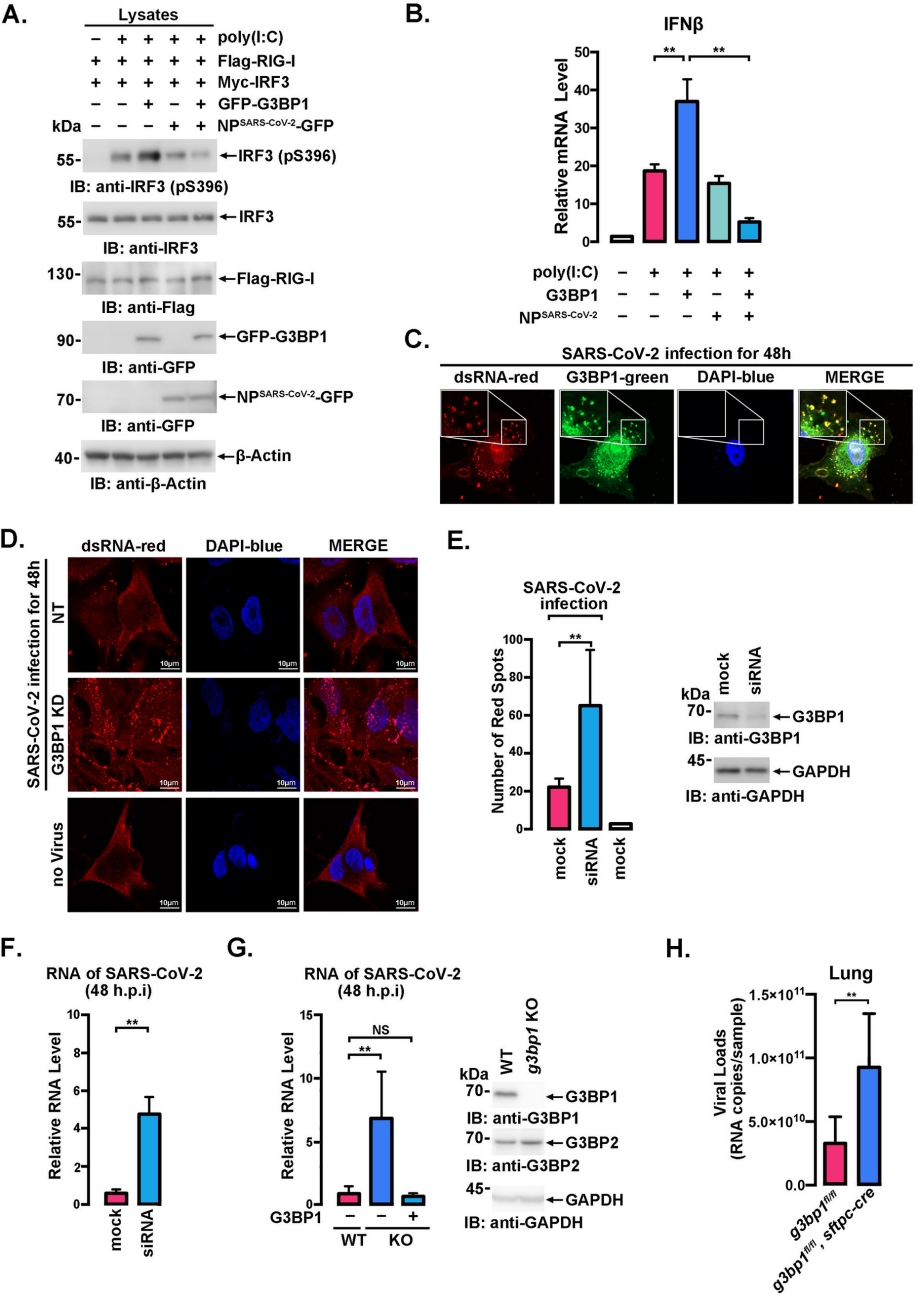
SARS-CoV-2 NP suppresses innate immunity by inhibiting G3BP1. (A) A549 cells cotransfected with the indicated plasmids were treated with or without 2 μg/mL poly(I·C) for 18 h, and immunoblot analysis was then performed with the indicated antibodies. (B) A549 cells cotransfected with the indicated plasmids were treated with or without 2.5 μg/mL poly(I·C) for 18 h. mRNA from the indicated cells was extracted to determine the mRNA transcript level of IFN-β via RT-PCR. All quantitative data are shown as the means ± SDs of three independent experiments. **, *P* < 0.05. (C and D) The subcellular localization of endogenous G3BP1 (green) and SARS-CoV-2 dsRNA (red) in wild-type G3BP1-knockdown A549 cells was detected by immunofluorescence staining using the indicated antibodies. The nuclei were stained with DAPI (blue). (E) The number of red spots in at least 10 cells shown in panel D was calculated, and the G3BP1-knockdown efficiency is shown. (F) RNA levels of SARS-CoV-2 in wild-type and G3BP1-knockdown A549 cells were analyzed by RT-PCR after 48 h of SARS-CoV-2 infection. All quantitative data are shown as the means ± SDs from three experiments. **, *P* < 0.05. (G) The RNA levels of SARS-CoV-2 in wild-type, G3BP1-knockout, and G3BP1-rescued A549 cells (right) were analyzed by RT-PCR after 48 h of SARS-CoV-2 infection. The G3BP1-knockout efficiency is shown. All quantitative data are shown as the means ± SDs from three experiments. **, *P* < 0.05. (H) WT mice (aged 8 to 10 weeks) and c-g3bp1-cKO mice (4 in each group) were intranasally infected with SARS-CoV-2 at 0.1 50% lethal dose (LD_50_), and the viral loads in the lungs of the infected mice were detected via RT-PCR. All quantitative data are shown as the means ± SDs (unpaired Student's *t* test). **, *P* < 0.01.

G3BP1 potentiates the RIG-I-related pathway via RNF125-mediated K48-linked polyubiquitination in response to viral infection, which results in IRF3 phosphorylation and increased IFN-β transcription (9). In agreement, IRF3 phosphorylation and IFN-β transcription were induced by G3BP1 in the context of poly(I·C) stimulation (Fig. 6A, lane 2), and G3BP1-potentiated IRF3 phosphorylation and IFN-β transcription were significantly suppressed by cotransfection of a plasmid expressing NP^SARS-CoV-2^ (Fig. 6A, lanes 3 and 5, and Fig. 6B); in addition, the ectopic expression of NP^SARS-CoV-2^ alone showed fewer but observable effects because the transfection efficiency was only ~30% in A549 cells, whereas the plasmids encoding G3BP1 and NP^SARS-CoV-2^ were usually equally delivered into the same cell (18). Notably, NP exerted weaker effects in the absence of G3BP1 overexpression (Fig. 6A, lanes 4 and 5, and Fig. 6B), which suggested that NP-G3BP1 or NP polymer-G3BP1 complexes may exert a stronger inhibitory effect on RIG-I activation than NP alone. These results collectively demonstrated that NP^SARS-CoV-2^ antagonizes G3BP1-mediated IRF3 phosphorylation and IFN-β production.

The effects of G3BP1 on SARS-CoV-2 replication were then evaluated through an analysis of double-stranded RNA (dsRNA), a presumed intermediate of viral replication, in SARS-CoV-2-infected A549 cells. The colocalization of G3BP1 with SARS-CoV-2 dsRNA was observed (Fig. 6C), and dsRNA formation was significantly increased by G3BP1 knockdown (Fig. 6D and E). The viral genomic RNA levels in culture supernatants were also significantly enhanced by G3BP1 knockdown, as demonstrated by quantitative real-time reverse transcription-PCR (RT-PCR) assays (Fig. 6F). Moreover, G3BP1 ablation in A549 cells (Fig. 6G, right) resulted in elevated viral replication, but this effect was markedly reversed by G3BP1 rescue (Fig. 6G, left). We then investigated the roles of G3BP1 in virus-infected G3BP1 conditional knockout mice (*g3bp1*-cKO, *g3bp1^fl/fl^*, *Sftpc*-Cre), in which G3BP1 was specifically knocked out in type II alveolar cells because complete G3BP1 knockout led to neonatal death (19). The lungs of cKO mice showed significantly higher viral loads at 3 days postintranasal infection with a mouse-adapted SARS-CoV-2 virus than those of their wild-type littermates (Fig. 6H). Together, these findings demonstrate that G3BP1 plays important roles in immunity against SARS-CoV-2 infection.

## DISCUSSION

Several recent studies have shown that SARS-CoV-2 NP impairs SG formation to promote the viral replication of SARS-CoV-2 via liquid-liquid phase separation (13, 16, 17, 20, 21). However, more information about the interactions between NP and G3BPs and more data from live virus-infected cells, mice, or even patients with COVID-19 need to be further investigated.

Compared with the published work, our study presents some novel findings. First, the interaction of NP with G3BPs was assessed in detail via surface plasmon resonance (SPR) analysis, and a strong, direct interaction with a *K_D_* at 10^−8^ to 10^−7^ M (Fig. 1I) was detected. Second, the results provide the first demonstration of the interaction between NP and G3BPs in the lung tissue of patients with COVID-19 and mouse-adapted SARS-CoV-2-infected mice (Fig. 2). Third, G3BP1 was found to play vital roles in SARS-CoV-2 infection, as shown using *g3bp1-*cKO mice, in which G3BP1 is specifically knocked out in type II alveolar cells (Fig. 6H). Finally, all NPs of all the tested coronaviruses, including SARS-CoV-2, SARS-CoV, MERS-CoV, and HCoV-OC43, were found to interact with G3BP (Fig. 1B to F), which suggested that the suppression of G3BPs-related host innate immunity might be a general or conserved strategy used by coronaviruses. Together, these data provide in-depth evidence showing the mechanism underlying NP-related SARS-CoV-2 pathogenesis through G3BPs.

In our study, we revealed the binding site between G3BP1 and NP. Interestingly, aa 1 to 142 of G3BP1 and aa 255 to 364 of NP, involved in G3BP1-NP interaction (Fig. 3B through D), were both reported to be responsible for homodimerization (14, 15). In accordance with the findings that N-ARM was necessary for SARS-CoV-2 N protein aggregation and condensation into SG (22), N-terminal deletion mutants of NP (Δ1-180 or Δ1-43) were demonstrated to decrease G3BP1 association significantly (Fig. 3C and D), which suggested that the N-ARM was involved in NP-G3BPs association, possibly by inducing conformation changes. Considering that the receptor-binding domain (RBD, which binds RNA) of G3BPs and the N-terminal domain (NTD) of NP (which binds viral genomic RNA) were not involved in the interaction, the CTD of NP may facilitate the RNA-contributed association because it could also bind RNA (23, 24). Moreover, consistent with the RNase A-digested association between NP^SARS-CoV-2^-Myc and Flag-G3BPs (Fig. 4A), the interactions between truncated G3BP1 and NP or between G3BP1/G3BP2 and truncated NP were not completely disrupted by RNase A digestion (Fig. 4B and C). The similar performance of the binding of G3BP mutants in the presence or absence of RNase A indicated that both the existence and spatial conformation of RNA may contribute to the association, but the exact underlying mechanism merits further investigation.

Studies have reported that SARS-CoV-2 infection impairs the IFN response (25, 26), and the expression of G3BP1 results in RIG-I activation by restricting the RNF125-mediated K48 ubiquitination and proteasomal degradation of RIG-I (9). In agreement with these findings, we found that G3BP1 could potentiate the phosphorylation of S396 in IRF3 (Fig. 6A), which plays crucial roles in the RIG-I signaling cascade, and this phosphorylation resulted in increased IFN production (27, 28). NP reportedly interacts with RIG-I and represses the IFN-β-related pathway (3, 5). Our study revealed that NP compromised the inhibition of IRF3 phosphorylation, probably due to the low transfection efficiency in A549 cells. Interestingly, more extensive inhibition was observed in cells ectopically expressing both NP and G3BP1 than in cells expressing only NP (Fig. 6A and B). These results suggested that NP^SARS-CoV-2^ overexpression, which results in abundant NP^SARS-CoV-2^, particularly in the presence of G3BP1-uncombined NPs, might induce the recruitment of G3BP1-binding proteins during SG formation and thus negatively impact RIG-I activation via the formation of NP-G3BP1 or NP polymer-G3BP1 complexes.

Consequently, the viral dsRNA levels in cells infected with live SARS-CoV-2 were increased by G3BP1-specific siRNA (Fig. 6D and E). The viral genomic RNA levels were also potentiated by G3BP1 disruption, and the effect was markedly reversed by G3BP1 rescue (Fig. 6G). Moreover, higher viral loads were observed in the lungs of *g3bp1*-cKO mice than in those of wild-type mice (Fig. 6H). Although the mouse-adapted SARS-CoV-2 virus exhibits one amino acid change in the N protein (D128Y) (29), the mutation, which does not affect the binding domain, was unlikely to restrict the G3BP1 interaction. Given these findings, we concluded that G3BP1 is of vital importance for the restriction of SARS-CoV-2 replication.

In summary, through an *in vitro* assay and live SARS-CoV-2 virus infection, this study provides solid evidence that SARS-CoV-2 NP associates with G3BP1 and G3BP2 *in vitro* and *in vivo* and that NP^SARS-CoV-2^ could efficiently suppress G3BPs-mediated SG formation and potentiate viral infection by overcoming G3BP1-mediated antiviral innate immunity, and these findings provide insightful evidence for improving the understanding of highly contagious SARS-CoV-2.

## MATERIALS AND METHODS

### Ethics statement.

This study followed the recommendations detailed in the Guide for the Care and Use of Laboratory Animals of the National Institutes of Health. All mouse protocols were approved by the Institutional Animal Care and Use Committee (IACUC) of the Laboratory Animal Center, Academy of Military Medical Sciences, China.

### Mice.

*G3bp1^fl/fL^* mice and *Sftpc*-Cre mice of the C57BL/6J background were purchased from GemPharmatech. *G3bp1^fl/fL^*, *Sftpc*-Cre mice were generated by crossing mice bearing loxP-flanked *g3bp1* alleles (*g3bp1^fl/fl^*) with a transgenic mouse line expressing Cre recombinase under the control of a modified distal promoter of the gene encoding *Sftpc* (*Sftpc*-Cre), which was specifically expressed in type II alveolar cells. The genotypes were identified by PCR analysis with mouse tail-tip DNA. Female mice (18 to 22 g, aged 6 to 8 weeks) were used in the experiments.

All mouse experiments were approved by the Animal Center at the Academy of Military Medical Sciences.

### Cell culture and transfection.

293T cells were grown in Dulbecco’s modified Eagle’s medium (Gibco) supplemented with 10% heat-inactivated fetal bovine serum (FBS; Biological Industries), 100 IU/mL ampicillin, and 100 μg/mL streptomycin. A549 cells were grown in Ham's F-12K (Kaighn's) medium (Gibco). The cells were transfected with Lipofectamine 2000 (Thermo Fisher) or the TransIT-X2 dynamic delivery system (Mirus).

The A549 cells used for the SARS-CoV-2 infection study were transfected with an ACE2-expressing plasmid prior to infection.

### Virus.

SARS-CoV-2 and mouse-adapted SARS-CoV-2 have been described previously (12). All experiments involving infectious SARS-CoV-2 were performed in a biosafety level 3 (BSL3) containment laboratory and approved by the Animal Experiment Committee of Laboratory Animal Center, Beijing Institute of Microbiology and Epidemiology.

### Generation of gene-knockout cell lines via CRISPR/Cas9.

We used the pSpCas9(BB)-2A-Puro plasmid (Addgene; plasmid ID 48139) for targeted G3BP1 gene knockout. The sgRNAs (*g3bp1*-1, 5′-GCTCATGCCACGCTAAATGATGG-3′; *g3bp1*-2, 5′-AACGTTTGTCCTTGCTCCTGAGG-3′) were designed using the online tool developed by F. Zhang’s laboratory (http://crispr.mit.edu/). Plasmids carrying a specific single guide RNA (sgRNA) sequence were transfected into the parental cells (A549), and 24 h later, the cells were treated with 1 μg/mL puromycin (Life Technologies). Cell clones were selected for expanded culture followed by genomic sequencing identification and immunoblot analysis.

### Vectors and epitope tagging of proteins.

Flag-tagged G3BP1, G3BP2, NP^SARS-CoV-2^, and mutants of G3BP1 were expressed by cloning the appropriate gene into p3xFLAG-CMV (Invitrogen). The genes encoding G3BP2 and NP^SARS-CoV-2^ were inserted into the pCMV-Myc plasmid (Clontech). eGFP-tagged G3BP1, NP^SARS-CoV-2^, NP^SARS-CoV^, NP^MERS-CoV^, NP^HCoV-OC43^, and mutants of NP^SARS-CoV-2^ were prepared by cloning the genes into the pEGFP-N1 plasmid (Clontech).

### LC-MS/MS analysis.

Anti-Flag antibody immunoprecipitates prepared from Flag-NP^SARS-CoV^-transfected 293FT cell lysates were resolved via SDS-PAGE, and the protein bands were then excised. After adequate digestion with chymotrypsin, LC-electrospray ionization-MS/MS-resolved peptides were analyzed using a quadrupole time of flight (Q-TOF) 2 MS system (MicroMass), and the data were compared against Swiss-Prot using the Mascot search engine (http://www.matrixscience.com) for peptide identification. The protein score calculated using Mascot was derived from ion scores as a nonprobabilistic basis for the ranking of protein hits. The ion scores were calculated as −10 × log(*P*), where *P* is the probability that the observed match is a random event. Individual ion scores higher than 43 indicate identity or extensive homology (*P* < 0.05). The exclusive spectrum counts were then analyzed, and the detected protein coverage of the total sample was calculated (see Table S1 in the supplemental material).

### Immunoprecipitation and immunoblotting.

Cell lysates were prepared in lysis buffer containing 1% Nonidet P-40. Soluble proteins were immunoprecipitated using anti-G3BP1 (catalog no. 61559; Cell Signaling Technology), anti-G3BP2 (catalog no. 16276-1-AP; Proteintech), anti-Myc (catalog no. E6654; Sigma-Aldrich), anti-Flag (catalog no. A2220; Sigma-Aldrich), and anti-mouse IgG (catalog no. A0919; Sigma-Aldrich) antibodies. An aliquot of the total lysate (5% [vol/vol]) was included as a control. Immunoblotting was performed with horseradish peroxidase (HRP)-conjugated anti-GFP (catalog no. SC-8334; Santa Cruz), HRP-conjugated anti-Myc (catalog no. SAB4200742; Sigma-Aldrich), HRP-conjugated anti-Flag (catalog no. A8592; Sigma-Aldrich), anti-G3BP1, anti-G3BP2, and anti-His (catalog no. SC-8036; Santa Cruz) antibodies. The antigen-antibody complexes were detected using an enhanced chemiluminescence (ECL) system (catalog no. 34095, Thermo Fisher). A PageRuler Western marker (catalog no. 6616, Thermo Fisher) was used as a molecular weight standard.

### Immunofluorescence.

A549 cells on glass coverslips were permeabilized with 0.2% Triton X-100 in PBS for 15 min and then fixed in paraformaldehyde (PFA; 4%) for 15 min. Antibodies against G3BP1, G3BP2, NP^SARS-CoV-2^ (catalog no. 40143-MM05; Sino Biological; and catalog no. DA027; Novoprotein), TIA1 (catalog no. PA5-19240; Invitrogen), and dsRNA (catalog no. J2; Scicons) were used as primary antibodies and visualized after cross-adsorbed secondary antibody binding. The localization of proteins was evaluated under a Zeiss LSM 800 confocal microscope.

### *In situ* PLA.

The associations between NP^SARS-CoV-2^ and G3BP1 or G3BP2 in lung sections from mice and COVID-19 patients were investigated by Duolink *in situ* PLA (Sigma-Aldrich). In brief, after sections were cut, the samples on glass coverslips were permeabilized with 0.2% Triton X-100 in phosphate-buffered saline (PBS) for 15 min and fixed in PFA (4%) for 15 min. Antibodies against G3BP1 or G3BP2 and NP^SARS-CoV-2^ were used according to the manufacturer's instructions for PLA. The protein-protein interactions were visualized as red fluorescent spots under a Zeiss LSM 800 confocal microscope.

### Reverse transcription-quantitative RT–PCR.

Total cellular RNA was extracted from cells (10^5^ cells) using the RNeasy minikit (catalog no. 74126; Qiagen), and total viral RNA was extracted from 2 mL of culture medium supernatant using the QIAamp viral RNA minikit (catalog no. 52906; Qiagen). RT-PCR was performed using the GoTaq 1-Step RT-qPCR system (catalog no. A6020, Promega) or novel coronavirus (2019-nCoV) nucleic acid detection kit (fluorescence RT-PCR). The primers used for gene detection are listed in Table 1, and GAPDH (glyceraldehyde-3-phosphate dehydrogenase) was used as a control for normalization of the RNA levels.

**TABLE 1:** Primer sequences for quantitative PCR, related to Fig. 6

Target^*a*^	qPCR primer sequence
G3BP1 (for mouse genotype identification)	5′-TGTCTGGATATTCCCCTGACTCAG-3′
	5′-TTCCCAGCATCCAGCTCCTAA-3′
SFTPC-CRE (for mouse genotype identification)	5′-ACCTTGTGAATGACCTCCAGG-3′
	5′-CATATAGACAAACGCACACCGGC-3′
NF (for viral load detection)	5′-GGGGAACTTCTCCTGCTAGAAT-3′
NR (for viral load detection)	5′-CAGACATTTTGCTCTCAAGCTG-3′
NP (for viral load detection)	5′-FAM-TTGCTGCTGCTTGACAGATT-TAMRA-3′

a NF, NP forward; NR, NP reverse; NP, NP Probe.

### Extraction of viral RNA and quantitative RT-PCR.

Tissue homogenates were prepared by the homogenization of whole tissue with an electric homogenizer for 300 s in 500 μL of TRIzol buffer. The homogenates were centrifuged at 12,000 rpm and 4°C for 10 min, and the supernatant was collected. Viral RNA was extracted using the QIAamp viral RNA minikit according to the manufacturer’s protocol. Viral RNA was quantified by quantitative reverse transcription-PCR targeting NP using premix Ex Taq (TaKaRa) with the following primers and probes (Table 1): NF, 5′-GGGGAACTTCTCCTGCTAGAAT-3′; NR, 5′-CAGACATTTTGCTCTCAAGCTG-3′; and NP, 5′-FAM-TTGCTGCTGCTTGACAGATT-TAMRA-3′.

### Protein purification for SPR.

The 6×His tag was fused to the N terminus of Flag-G3BP1/G3BP2 in the pcDNA3.1 plasmid. 293T cells were transfected with the indicated plasmids, and 48 h later, the cells were subjected to ultrasonic lysis. We purified 6×His-tagged proteins through Ni-nitrilotriacetic acid (NTA) column affinity chromatography, and they were analyzed by SDS-PAGE and immunoblotting.

### SPR assay.

The NP of SARS-CoV-2 (catalog no. 40588-V07E; Sino Biological) or SARS-CoV (catalog no. 40143-V08B, Sino Biological) was immobilized on CM5 sensor chips (Biacore; GE) at a level of ~2,000 response units (RUs) using a Biacore T200 (Biacore; GE) and running buffer composed of 0.01 M HEPES (pH 7.4), 0.15 M NaCl, 3 mM EDTA, and 0.05% Tween 20. Serial dilutions of G3BP1 and G3BP2 were flown through at concentrations ranging from 125 to 3.91 nM. The resulting data were fit to a 1:1 binding model using Biacore Evaluation Software (Biacore; GE).

### Statistical analysis.

Patches of SGs or dsRNA were analyzed using ImageJ software, and the boundary-clear patches were counted as individual SGs or dsRNAs. The mean values from at least three independent experiments were analyzed by Student's *t* test using GraphPad Prism software. Differences with a *P* value of <0.05 were considered significant.

### Data availability.

The genome sequences of IME-BJ05 (human clinical isolate) and MASCp6 (mouse-adapted SARS-CoV-2) have been deposited in the Genome Warehouse in National Genomics Data Center (https://ngdc.cncb.ac.cn/gwh/), BIG, CAS, with the accession nos. GWHACBB01000000.2 and GWHACFH00000000, respectively.
