# Supplementary material for: SARS-CoV-2 N Protein Antagonizes Stress Granule Assembly and IFN Production by Interacting with G3BPs to Facilitate Viral Replication
Source: J Virol. 2022 Jun 2;96(12):e00412-22. doi: 10.1128/jvi.00412-22 (PMC9215227; doi:10.1128/jvi.00412-22)
Supplement: Supplemental file 1 — Table S1. Download jvi.00412-22-s0001.pdf, PDF file, 0.5 MB [file jvi.00412-22-s0001.pdf]

Table S1: Data of the liquid chromatography (LC)-MS/MS analysis

↑ Family	M	DB	Accession	Score	Mass	Matches	Match(sig)	Sequences	Seq(sig)	emPAI	Description
2	1	swissprot_human	sp|Q13283|G3BP1_HUMAN	1317	52189	133	78	17	12	2.12	sp|Q13283|G3BP1_HUMAN Ras GTPase-activating protein-binding protein 1 OS=Homo sapiens OX=9606 GN=G3BP1 PE=1 SV=1
1	5	swissprot_human	sp|P11021|BIP_HUMAN	822	72402	75	52	32	25	3.89	sp|P11021|BIP_HUMAN Endoplasmic reticulum chaperone BiP OS=Homo sapiens OX=9606 GN=HSPA5 PE=1 SV=2
5	1	swissprot_human	sp|P52272|HNRPM_HUMAN	700	77749	84	48	43	35	7.47	sp|P52272|HNRPM_HUMAN Heterogeneous nuclear ribonucleoprotein M OS=Homo sapiens OX=9606 GN=HNRNPM PE=1 SV=3
1	6	swissprot_human	sp|P38646|GRP75_HUMAN	693	73920	66	41	34	27	3.73	sp|P38646|GRP75_HUMAN Stress-70 protein, mitochondrial OS=Homo

											sapiens OX=9606 GN=HSPA9 PE=1 SV=2
3	2	swissprot_human	sp|P35908|K22E_HUMAN	686	65678	55	37	33	26	4.38	sp|P35908|K22E_HUMAN Keratin, type II cytoskeletal 2 epidermal OS=Homo sapiens OX=9606 GN=KRT2 PE=1 SV=2
6	1	swissprot_human	sp|P27694|RFA1_HUMAN	606	68723	51	29	28	20	2.9	sp|P27694|RFA1_HUMAN Replication protein A 70 kDa DNA-binding subunit OS=Homo sapiens OX=9606 GN=RPA1 PE=1 SV=2
7	1	swissprot_human	sp|Q9NZI8|IF2B1_HUMAN	575	63783	62	31	27	20	2.79	sp|Q9NZI8|IF2B1_HUMAN Insulin-like growth factor 2 mRNA-binding protein 1 OS=Homo sapiens OX=9606 GN=IGF2BP1 PE=1 SV=2
4	2	swissprot_human	sp|P13645|K1C10_HUMAN	554	59020	47	29	23	19	3.22	sp|P13645|K1C10_HUMAN Keratin, type I cytoskeletal 10 OS=Homo sapiens

											OX=9606 GN=KRT10 PE=1 SV=6
2	2	swissprot_human	sp|Q9UN86|G3BP2_HUMAN	536	54145	98	44	20	15	2.51	sp|Q9UN86|G3BP2_HUMAN Ras GTPase-activating protein-binding protein 2 OS=Homo sapiens OX=9606 GN=G3BP2 PE=1 SV=2
8	1	swissprot_human	sp|P17844|DDX5_HUMAN	491	69618	47	32	32	26	3.61	sp|P17844|DDX5_HUMAN Probable ATP-dependent RNA helicase DDX5 OS=Homo sapiens OX=9606 GN=DDX5 PE=1 SV=1
9	1	swissprot_human	sp|P11940|PABP1_HUMAN	460	70854	70	36	34	24	3.49	sp|P11940|PABP1_HUMAN Polyadenylate-binding protein 1 OS=Homo sapiens OX=9606 GN=PABPC1 PE=1 SV=2
10	1	swissprot_human	sp|Q86YZ3|HORN_HUMAN	438	283140	16	13	13	11	0.18	sp|Q86YZ3|HORN_HUMAN Hornerin OS=Homo sapiens OX=9606 GN=HRNR PE=1 SV=2
4	3	swissprot_human	sp|P08779|K1C16_HUMAN	426	51578	23	19	17	15	2.73	sp|P08779|K1C16_HUMAN Keratin, type I

											cytoskeletal 16 OS=Homo sapiens OX=9606 GN=KRT16 PE=1 SV=4
11	1	swissprot_human	sp|P61978|HNRPK_HUMAN	409	51230	37	21	16	8	1.11	sp|P61978|HNRPK_HUMAN Heterogeneous nuclear ribonucleoprotein K OS=Homo sapiens OX=9606 GN=HNRNPK PE=1 SV=1
12	1	swissprot_human	sp|Q96PK6|RBM14_HUMAN	392	69620	27	18	22	15	1.5	sp|Q96PK6|RBM14_HUMAN RNA-binding protein 14 OS=Homo sapiens OX=9606 GN=RBM14 PE=1 SV=2
8	2	swissprot_human	sp|Q92841|DDX17_HUMAN	373	80906	28	18	21	15	1.2	sp|Q92841|DDX17_HUMAN Probable ATP-dependent RNA helicase DDX17 OS=Homo sapiens OX=9606 GN=DDX17 PE=1 SV=2
13	1	swissprot_human	sp|O76021|RL1D1_HUMAN	373	55167	44	24	28	19	3.32	sp|O76021|RL1D1_HUMAN Ribosomal L1 domain-containing protein 1 OS=Homo

											sapiens OX=9606 GN=RSL1D1 PE=1 SV=3
4	4	swissprot_human	sp|P02533|K1C14_HUMAN	350	51872	22	18	15	13	2.15	sp|P02533|K1C14_HUMAN Keratin, type I cytoskeletal 14 OS=Homo sapiens OX=9606 GN=KRT14 PE=1 SV=4
14	1	swissprot_human	sp|P19338|NUCL_HUMAN	343	76625	30	18	22	15	1.18	sp|P19338|NUCL_HUMAN Nucleolin OS=Homo sapiens OX=9606 GN=NCL PE=1 SV=3
15	1	swissprot_human	sp|Q07666|KHDR1_HUMAN	339	48311	29	16	15	10	1.63	sp|Q07666|KHDR1_HUMAN KH domain-containing, RNA-binding, signal transduction-associated protein 1 OS=Homo sapiens OX=9606 GN=KHDRBS1 PE=1 SV=1
16	1	swissprot_human	sp|O60506|HNRPQ_HUMAN	332	69788	30	22	21	15	1.65	sp|O60506|HNRPQ_HUMAN Heterogeneous nuclear ribonucleoprotein Q OS=Homo sapiens

											OX=9606 GN=SYNCRIP PE=1 SV=2
3	3	swissprot_human	sp|P02538|K2C6A_HUMAN	315	60293	32	19	26	17	2.32	sp|P02538|K2C6A_HUMAN Keratin, type II cytoskeletal 6A OS=Homo sapiens OX=9606 GN=KRT6A PE=1 SV=3
17	1	swissprot_human	sp|O14744|ANM5_HUMAN	311	73322	47	24	21	15	1.39	sp|O14744|ANM5_HUMAN Protein arginine N-methyltransferase 5 OS=Homo sapiens OX=9606 GN=PRMT5 PE=1 SV=4
3	4	swissprot_human	sp|P04259|K2C6B_HUMAN	310	60315	35	21	26	17	2.32	sp|P04259|K2C6B_HUMAN Keratin, type II cytoskeletal 6B OS=Homo sapiens OX=9606 GN=KRT6B PE=1 SV=5
9	2	swissprot_human	sp|Q13310|PABP4_HUMAN	307	71080	43	25	30	21	2.52	sp|Q13310|PABP4_HUMAN Polyadenylate-binding protein 4 OS=Homo sapiens OX=9606 GN=PABPC4 PE=1 SV=1

16	2	swissprot_human	sp|O43390|HNRPR_HUMAN	302	71184	23	17	16	13	1.31	sp|O43390|HNRPR_HUMAN Heterogeneous nuclear ribonucleoprotein R OS=Homo sapiens OX=9606 GN=HNRNPR PE=1 SV=1
3	5	swissprot_human	sp|P13647|K2C5_HUMAN	288	62568	28	18	25	17	2.17	sp|P13647|K2C5_HUMAN Keratin, type II cytoskeletal 5 OS=Homo sapiens OX=9606 GN=KRT5 PE=1 SV=3
18	1	swissprot_human	sp|P10809|CH60_HUMAN	277	61187	25	12	20	10	1	sp|P10809|CH60_HUMAN 60 kDa heat shock protein, mitochondrial OS=Homo sapiens OX=9606 GN=HSPD1 PE=1 SV=2
8	3	swissprot_human	sp|O00571|DDX3X_HUMAN	267	73597	27	16	23	15	1.38	sp|O00571|DDX3X_HUMAN ATP-dependent RNA helicase DDX3X OS=Homo sapiens OX=9606 GN=DDX3X PE=1 SV=3
19	1	swissprot_human	sp|Q00839|HNRPU_HUMAN	225	91269	25	14	18	11	0.67	sp|Q00839|HNRPU_HUMAN Heterogeneous nuclear ribonucleoprotein

											U OS=Homo sapiens OX=9606 GN=HNRNPU PE=1 SV=6
7	2	swissprot_human	sp|O00425|IF2B3_HUMAN	210	64008	19	13	12	9	0.82	sp|O00425|IF2B3_HUMAN Insulin-like growth factor 2 mRNA-binding protein 3 OS=Homo sapiens OX=9606 GN=IGF2BP3 PE=1 SV=2
20	1	swissprot_human	sp|Q9BZE4|NOG1_HUMAN	199	74317	20	11	18	10	0.77	sp|Q9BZE4|NOG1_HUMAN Nucleolar GTP-binding protein 1 OS=Homo sapiens OX=9606 GN=GTPBP4 PE=1 SV=3
7	3	swissprot_human	sp|Q9Y6M1|IF2B2_HUMAN	194	66195	15	10	12	9	0.78	sp|Q9Y6M1|IF2B2_HUMAN Insulin-like growth factor 2 mRNA-binding protein 2 OS=Homo sapiens OX=9606 GN=IGF2BP2 PE=1 SV=2
21	1	swissprot_human	sp|O00541|PESC_HUMAN	192	68359	14	8	14	8	0.64	sp|O00541|PESC_HUMAN Pescadillo homolog OS=Homo sapiens

											OX=9606 GN=PES1 PE=1 SV=1
22	1	swissprot_human	sp|P14866|HNRPL_HUMAN	184	64720	14	9	12	8	0.69	sp|P14866|HNRPL_HUMAN Heterogeneous nuclear ribonucleoprotein L OS=Homo sapiens OX=9606 GN=HNRNPL PE=1 SV=2
23	1	swissprot_human	sp|Q9NVI7|ATD3A_HUMAN	179	71610	23	15	20	13	1.17	sp|Q9NVI7|ATD3A_HUMAN ATPase family AAA domain-containing protein 3A OS=Homo sapiens OX=9606 GN=ATAD3A PE=1 SV=2
24	1	swissprot_human	sp|Q9BZI7|REN3B_HUMAN	176	57841	20	10	17	8	0.8	sp|Q9BZI7|REN3B_HUMAN Regulator of nonsense transcripts 3B OS=Homo sapiens OX=9606 GN=UPF3B PE=1 SV=1
25	1	swissprot_human	sp|Q9Y2W1|TR150_HUMAN	176	108658	23	14	21	12	0.6	sp|Q9Y2W1|TR150_HUMAN Thyroid hormone receptor-associated protein 3 OS=Homo sapiens OX=9606

											GN=THRAP3 PE=1 SV=2
26	1	swissprot_human	sp|Q07065|CKAP4_HUMAN	161	66097	15	11	14	10	0.9	sp|Q07065|CKAP4_HUMAN Cytoskeleton-associated protein 4 OS=Homo sapiens OX=9606 GN=CKAP4 PE=1 SV=2
27	1	swissprot_human	sp|P35637|FUS_HUMAN	161	53622	25	11	10	7	0.88	sp|P35637|FUS_HUMAN RNA-binding protein FUS OS=Homo sapiens OX=9606 GN=FUS PE=1 SV=1
3	6	swissprot_human	sp|Q7Z794|K2C1B_HUMAN	155	62149	12	6	7	4	0.31	sp|Q7Z794|K2C1B_HUMAN Keratin, type II cytoskeletal 1b OS=Homo sapiens OX=9606 GN=KRT77 PE=2 SV=3
23	2	swissprot_human	sp|Q5T9A4|ATD3B_HUMAN	148	73098	17	10	16	10	0.79	sp|Q5T9A4|ATD3B_HUMAN ATPase family AAA domain-containing protein 3B OS=Homo sapiens OX=9606 GN=ATAD3B PE=1 SV=1

28	1	swissprot_human	sp|P17987|TCPA_HUMAN	147	60819	17	10	13	8	0.75	sp|P17987|TCPA_HUMAN T-complex protein 1 subunit alpha OS=Homo sapiens OX=9606 GN=TCP1 PE=1 SV=1
29	1	swissprot_human	sp|Q9UHX1|PUF60_HUMAN	147	60009	11	8	9	8	0.76	sp|Q9UHX1|PUF60_HUMAN Poly(U)-binding-splicing factor PUF60 OS=Homo sapiens OX=9606 GN=PUF60 PE=1 SV=1
30	1	swissprot_human	sp|Q9UHB9|SRP68_HUMAN	146	71199	13	10	13	10	0.82	sp|Q9UHB9|SRP68_HUMAN Signal recognition particle subunit SRP68 OS=Homo sapiens OX=9606 GN=SRP68 PE=1 SV=2
31	1	swissprot_human	sp|Q9BVP2|GNL3_HUMAN	145	62468	20	12	16	11	1.11	sp|Q9BVP2|GNL3_HUMAN Guanine nucleotide-binding protein-like 3 OS=Homo sapiens OX=9606 GN=GNL3 PE=1 SV=2
25	2	swissprot_human	sp|Q9NYF8|BCLF1_HUMAN	144	106173	24	11	19	10	0.49	sp|Q9NYF8|BCLF1_HUMAN Bcl-2-associated transcription factor 1

											OS=Homo sapiens OX=9606 GN=BCLAF1 PE=1 SV=2
32	1	swissprot_human	sp|Q9NZM5|NOP53_HUMAN	137	54470	10	7	9	7	0.73	sp|Q9NZM5|NOP53_HUMAN Ribosome biogenesis protein NOP53 OS=Homo sapiens OX=9606 GN=NOP53 PE=1 SV=2
33	1	swissprot_human	sp|P20700|LMNB1_HUMAN	136	66653	13	8	12	8	0.67	sp|P20700|LMNB1_HUMAN Lamin-B1 OS=Homo sapiens OX=9606 GN=LMNB1 PE=1 SV=2
34	1	swissprot_human	sp|Q14498|RBM39_HUMAN	134	59628	12	8	10	7	0.77	sp|Q14498|RBM39_HUMAN RNA-binding protein 39 OS=Homo sapiens OX=9606 GN=RBM39 PE=1 SV=2
31	2	swissprot_human	sp|Q9NVN8|GNL3L_HUMAN	130	66216	10	8	10	8	0.67	sp|Q9NVN8|GNL3L_HUMAN Guanine nucleotide-binding protein-like 3-like protein OS=Homo sapiens OX=9606 GN=GNL3L PE=1 SV=1

35	1	swissprot_human	sp|P51114|FXR1_HUMAN	127	70020	16	7	14	6	0.44	sp|P51114|FXR1_HUMAN Fragile X mental retardation syndrome-related protein 1 OS=Homo sapiens OX=9606 GN=FXR1 PE=1 SV=3
36	1	swissprot_human	sp|P26368|U2AF2_HUMAN	126	53809	11	8	7	5	0.61	sp|P26368|U2AF2_HUMAN Splicing factor U2AF 65 kDa subunit OS=Homo sapiens OX=9606 GN=U2AF2 PE=1 SV=4
37	1	swissprot_human	sp|Q13573|SNW1_HUMAN	123	61514	14	8	12	8	0.74	sp|Q13573|SNW1_HUMAN SNW domain-containing protein 1 OS=Homo sapiens OX=9606 GN=SNW1 PE=1 SV=1
38	1	swissprot_human	sp|O14654|IRS4_HUMAN	117	134711	13	8	12	7	0.25	sp|O14654|IRS4_HUMAN Insulin receptor substrate 4 OS=Homo sapiens OX=9606 GN=IRS4 PE=1 SV=1
39	1	swissprot_human		117	56363	13	8	9	7	0.69	sp|Q13610|PWP1_HUMAN Periodic tryptophan

			sp|Q13610|PWP1_HUMAN								protein 1 homolog OS=Homo sapiens OX=9606 GN=PWP1 PE=1 SV=1
40	1	swissprot_human	sp|P55081|MFAP1_HUMAN	116	51927	19	7	16	5	0.5	sp|P55081|MFAP1_HUMAN Microfibrillar-associated protein 1 OS=Homo sapiens OX=9606 GN=MFAP1 PE=1 SV=2
35	2	swissprot_human	sp|Q06787|FMR1_HUMAN	110	71473	15	9	12	8	0.61	sp|Q06787|FMR1_HUMAN Synaptic functional regulator FMR1 OS=Homo sapiens OX=9606 GN=FMR1 PE=1 SV=1
41	1	swissprot_human	sp|Q9NY93|DDX56_HUMAN	109	62007	6	5	6	5	0.41	sp|Q9NY93|DDX56_HUMAN Probable ATP-dependent RNA helicase DDX56 OS=Homo sapiens OX=9606 GN=DDX56 PE=1 SV=1
42	1	swissprot_human	sp|O75688|PPM1B_HUMAN	109	53180	13	7	10	7	0.75	sp|O75688|PPM1B_HUMAN Protein phosphatase 1B OS=Homo sapiens

											OX=9606 GN=PPM1B PE=1 SV=1
43	1	swissprot_human	sp|P16402|H13_HUMAN	108	22336	33	4	7	4	1.12	sp|P16402|H13_HUMAN Histone H1.3 OS=Homo sapiens OX=9606 GN=H1-3 PE=1 SV=2
44	1	swissprot_human	sp|A0A075B6S2|KVD29_HUMAN	108	13249	6	3	2	1	0.37	sp|A0A075B6S2|KVD29_HUMAN Immunoglobulin kappa variable 2D-29 OS=Homo sapiens OX=9606 GN=IGKV2D-29 PE=3 SV=1
45	1	swissprot_human	sp|Q8N1G4|LRC47_HUMAN	105	64004	7	3	6	3	0.22	sp|Q8N1G4|LRC47_HUMAN Leucine-rich repeat-containing protein 47 OS=Homo sapiens OX=9606 GN=LRRC47 PE=1 SV=1
46	1	swissprot_human	sp|P04843|RPN1_HUMAN	104	68641	8	3	7	3	0.2	sp|P04843|RPN1_HUMAN Dolichyl-diphosphooligosaccharide--protein glycosyltransferase subunit 1 OS=Homo

											sapiens OX=9606 GN=RPN1 PE=1 SV=1
47	1	swissprot_human	sp|P49368|TCPG_HUMAN	104	61066	11	6	11	6	0.52	sp|P49368|TCPG_HUMAN T-complex protein 1 subunit gamma OS=Homo sapiens OX=9606 GN=CCT3 PE=1 SV=4
48	1	swissprot_human	sp|P08621|RU17_HUMAN	103	51583	22	13	16	10	1.28	sp|P08621|RU17_HUMAN U1 small nuclear ribonucleoprotein 70 kDa OS=Homo sapiens OX=9606 GN=SNRNP70 PE=1 SV=2
49	1	swissprot_human	sp|Q86UE4|LYRIC_HUMAN	102	63856	12	4	12	4	0.31	sp|Q86UE4|LYRIC_HUMAN Protein LYRIC OS=Homo sapiens OX=9606 GN=MTDH PE=1 SV=2
50	1	swissprot_human	sp|O95831|AIFM1_HUMAN	101	67144	13	7	11	7	0.56	sp|O95831|AIFM1_HUMAN Apoptosis-inducing factor 1, mitochondrial OS=Homo sapiens OX=9606 GN=AIFM1 PE=1 SV=1

51	1	swissprot_human	sp|Q6UN15|FIP1_HUMAN	101	66601	4	3	4	3	0.21	sp|Q6UN15|FIP1_HUMAN Pre-mRNA 3'-end-processing factor FIP1 OS=Homo sapiens OX=9606 GN=FIP1L1 PE=1 SV=1
52	1	swissprot_human	sp|Q96I24|FUBP3_HUMAN	99	61944	11	9	10	8	0.73	sp|Q96I24|FUBP3_HUMAN Far upstream element-binding protein 3 OS=Homo sapiens OX=9606 GN=FUBP3 PE=1 SV=2
3	7	swissprot_human	sp|Q7RTS7|K2C74_HUMAN	96	58229	7	5	6	5	0.44	sp|Q7RTS7|K2C74_HUMAN Keratin, type II cytoskeletal 74 OS=Homo sapiens OX=9606 GN=KRT74 PE=1 SV=2
53	1	swissprot_human	sp|Q5SSJ5|HP1B3_HUMAN	96	61454	13	5	11	5	0.41	sp|Q5SSJ5|HP1B3_HUMAN Heterochromatin protein 1-binding protein 3 OS=Homo sapiens OX=9606 GN=HP1BP3 PE=1 SV=1
54	1	swissprot_human		95	51834	9	3	8	2	0.18	sp|O95232|LC7L3_HUMAN Luc7-like protein 3

			sp|O95232|LC7L3_HUMAN								OS=Homo sapiens OX=9606 GN=LUC7L3 PE=1 SV=2
55	1	swissprot_human	sp|Q9UBU9|NXF1_HUMAN	93	70652	16	5	16	5	0.35	sp|Q9UBU9|NXF1_HUMAN Nuclear RNA export factor 1 OS=Homo sapiens OX=9606 GN=NXF1 PE=1 SV=1
27	2	swissprot_human	sp|Q92804|RBP56_HUMAN	91	62021	11	5	5	3	0.23	sp|Q92804|RBP56_HUMAN TATA-binding protein-associated factor 2N OS=Homo sapiens OX=9606 GN=TAF15 PE=1 SV=1
56	1	swissprot_human	sp|Q96RR1|PEO1_HUMAN	90	77619	10	5	8	5	0.32	sp|Q96RR1|PEO1_HUMAN Twinkle protein, mitochondrial OS=Homo sapiens OX=9606 GN=TWNK PE=1 SV=1
57	1	swissprot_human	sp|Q96GA3|LTV1_HUMAN	88	55049	6	3	5	3	0.26	sp|Q96GA3|LTV1_HUMAN Protein LTV1 homolog OS=Homo sapiens OX=9606 GN=LTV1 PE=1 SV=1
58	1	swissprot_human		85	60089	9	5	9	5	0.42	sp|P48643|TCPE_HUMAN T-complex protein 1

			sp|P48643|TCPE_HUMAN								subunit epsilon OS=Homo sapiens OX=9606 GN=CCT5 PE=1 SV=1
59	1	swissprot_human	sp|Q9UJV9|DDX41_HUMAN	85	70477	14	7	12	6	0.44	sp|Q9UJV9|DDX41_HUMAN Probable ATP-dependent RNA helicase DDX41 OS=Homo sapiens OX=9606 GN=DDX41 PE=1 SV=2
60	1	swissprot_human	sp|P54577|SYYC_HUMAN	85	59448	6	5	6	5	0.43	sp|P54577|SYYC_HUMAN Tyrosine--tRNA ligase, cytoplasmic OS=Homo sapiens OX=9606 GN=YARS PE=1 SV=4
61	1	swissprot_human	sp|Q16630|CPSF6_HUMAN	82	59344	8	5	5	4	0.33	sp|Q16630|CPSF6_HUMAN Cleavage and polyadenylation specificity factor subunit 6 OS=Homo sapiens OX=9606 GN=CPSF6 PE=1 SV=2
62	1	swissprot_human	sp|Q9Y2R4|DDX52_HUMAN	82	67798	6	3	5	3	0.21	sp|Q9Y2R4|DDX52_HUMAN Probable ATP-dependent RNA helicase DDX52 OS=Homo

											sapiens OX=9606 GN=DDX52 PE=1 SV=3
63	1	swissprot_human	sp|P40227|TCPZ_HUMAN	81	58444	13	6	13	6	0.55	sp|P40227|TCPZ_HUMAN T-complex protein 1 subunit zeta OS=Homo sapiens OX=9606 GN=CCT6A PE=1 SV=3
64	1	swissprot_human	sp|Q8ND56|LS14A_HUMAN	80	50727	18	5	11	4	0.4	sp|Q8ND56|LS14A_HUMAN Protein LSM14 homolog A OS=Homo sapiens OX=9606 GN=LSM14A PE=1 SV=3
65	1	swissprot_human	sp|Q15637|SF01_HUMAN	80	68514	10	5	8	5	0.36	sp|Q15637|SF01_HUMAN Splicing factor 1 OS=Homo sapiens OX=9606 GN=SF1 PE=1 SV=4
66	1	swissprot_human	sp|P60709|ACTB_HUMAN	80	42052	5	4	5	4	0.5	sp|P60709|ACTB_HUMAN Actin, cytoplasmic 1 OS=Homo sapiens OX=9606 GN=ACTB PE=1 SV=1
67	1	swissprot_human	sp|Q9Y5J1|UTP18_HUMAN	79	62421	7	5	5	4	0.31	sp|Q9Y5J1|UTP18_HUMAN U3 small nucleolar RNA-associated protein 18 homolog OS=Homo

											sapiens OX=9606 GN=UTP18 PE=1 SV=3
68	1	swissprot_human	sp|Q86U38|NOP9_HUMAN	78	70136	4	2	4	2	0.13	sp|Q86U38|NOP9_HUMAN Nucleolar protein 9 OS=Homo sapiens OX=9606 GN=NOP9 PE=1 SV=1
69	1	swissprot_human	sp|Q08211|DHX9_HUMAN	76	142181	13	7	12	6	0.2	sp|Q08211|DHX9_HUMAN ATP-dependent RNA helicase A OS=Homo sapiens OX=9606 GN=DHX9 PE=1 SV=4
70	1	swissprot_human	sp|Q5T3I0|GPTC4_HUMAN	72	50579	13	4	11	3	0.29	sp|Q5T3I0|GPTC4_HUMAN G patch domain-containing protein 4 OS=Homo sapiens OX=9606 GN=GPATCH4 PE=1 SV=2
71	1	swissprot_human	sp|Q9H5H4|ZN768_HUMAN	71	61445	9	3	9	3	0.23	sp|Q9H5H4|ZN768_HUMAN Zinc finger protein 768 OS=Homo sapiens OX=9606 GN=ZNF768 PE=1 SV=2
72	1	swissprot_human	sp|O76094|SRP72_HUMAN	71	75130	12	5	11	5	0.33	sp|O76094|SRP72_HUMAN Signal recognition particle subunit SRP72

											OS=Homo sapiens OX=9606 GN=SRP72 PE=1 SV=3
73	1	swissprot_human	sp|P54136|SYRC_HUMAN	70	76129	9	4	8	4	0.25	sp|P54136|SYRC_HUMAN Arginine--tRNA ligase, cytoplasmic OS=Homo sapiens OX=9606 GN=RARS PE=1 SV=2
74	1	swissprot_human	sp|Q5BKZ1|ZN326_HUMAN	68	65955	8	6	7	5	0.38	sp|Q5BKZ1|ZN326_HUMAN DBIRD complex subunit ZNF326 OS=Homo sapiens OX=9606 GN=ZNF326 PE=1 SV=2
75	1	swissprot_human	sp|P36578|RL4_HUMAN	62	47953	6	2	6	2	0.19	sp|P36578|RL4_HUMAN 60S ribosomal protein L4 OS=Homo sapiens OX=9606 GN=RPL4 PE=1 SV=5
76	1	swissprot_human	sp|Q8WWY3|PRP31_HUMAN	62	55649	8	3	7	3	0.26	sp|Q8WWY3|PRP31_HUMAN U4/U6 small nuclear ribonucleoprotein Prp31 OS=Homo sapiens OX=9606 GN=PRPF31 PE=1 SV=2

77	1	swissprot_human	sp|Q32NB8|PGPS1_HUMAN	61	63147	1	1	1	1	0.07	sp|Q32NB8|PGPS1_HUMAN CDP-diacylglycerol--glycerol-3-phosphate 3-phosphatidyltransferase, mitochondrial OS=Homo sapiens OX=9606 GN=PGS1 PE=2 SV=1
78	1	swissprot_human	sp|Q9Y6Y0|NS1BP_HUMAN	61	72937	7	6	7	6	0.42	sp|Q9Y6Y0|NS1BP_HUMAN Influenza virus NS1A-binding protein OS=Homo sapiens OX=9606 GN=IVNS1ABP PE=1 SV=3
79	1	swissprot_human	sp|Q15750|TAB1_HUMAN	61	54895	3	2	3	2	0.17	sp|Q15750|TAB1_HUMAN TGF-beta-activated kinase 1 and MAP3K7-binding protein 1 OS=Homo sapiens OX=9606 GN=TAB1 PE=1 SV=1
80	1	swissprot_human	sp|O76031|CLPX_HUMAN	59	69922	8	4	8	4	0.28	sp|O76031|CLPX_HUMAN ATP-dependent Clp protease ATP-binding subunit clpX-like, mitochondrial OS=Homo

											sapiens OX=9606 GN=CLPX PE=1 SV=2
81	1	swissprot_human	sp|Q08170|SRSF4_HUMAN	58	56759	9	4	7	4	0.35	sp|Q08170|SRSF4_HUMAN Serine/arginine-rich splicing factor 4 OS=Homo sapiens OX=9606 GN=SRSF4 PE=1 SV=2
82	1	swissprot_human	sp|Q8WXF1|PSPC1_HUMAN	57	58820	7	3	7	3	0.24	sp|Q8WXF1|PSPC1_HUMAN Paraspeckle component 1 OS=Homo sapiens OX=9606 GN=PSPC1 PE=1 SV=1
83	1	swissprot_human	sp|Q8NC51|PAIRB_HUMAN	56	44995	4	2	4	2	0.21	sp|Q8NC51|PAIRB_HUMAN Plasminogen activator inhibitor 1 RNA-binding protein OS=Homo sapiens OX=9606 GN=SERBP1 PE=1 SV=2
84	1	swissprot_human	sp|Q9NR30|DDX21_HUMAN	56	87804	5	3	5	3	0.16	sp|Q9NR30|DDX21_HUMAN Nucleolar RNA helicase 2 OS=Homo sapiens OX=9606 GN=DDX21 PE=1 SV=5

85	1	swissprot_human	sp|Q14258|TRI25_HUMAN	51	72581	6	3	4	3	0.19	sp|Q14258|TRI25_HUMAN E3 ubiquitin/ISG15 ligase TRIM25 OS=Homo sapiens OX=9606 GN=TRIM25 PE=1 SV=2
86	1	swissprot_human	sp|Q9UK59|DBR1_HUMAN	49	62144	5	1	3	1	0.07	sp|Q9UK59|DBR1_HUMAN Lariat debranching enzyme OS=Homo sapiens OX=9606 GN=DBR1 PE=1 SV=2
87	1	swissprot_human	sp|Q96SI9|STRBP_HUMAN	49	74290	10	5	9	5	0.33	sp|Q96SI9|STRBP_HUMAN Spermatid perinuclear RNA-binding protein OS=Homo sapiens OX=9606 GN=STRBP PE=1 SV=1
88	1	swissprot_human	sp|Q9BZZ5|API5_HUMAN	49	59310	10	5	10	5	0.43	sp|Q9BZZ5|API5_HUMAN Apoptosis inhibitor 5 OS=Homo sapiens OX=9606 GN=API5 PE=1 SV=3
89	1	swissprot_human	sp|Q8N3Y1|FBXW8_HUMAN	49	67865	3	2	3	2	0.13	sp|Q8N3Y1|FBXW8_HUMAN F-box/WD repeat-containing protein 8 OS=Homo sapiens

											OX=9606 GN=FBXW8 PE=1 SV=2
90	1	swissprot_human	sp|O60832|DKC1_HUMAN	49	58094	9	2	9	2	0.16	sp|O60832|DKC1_HUMAN H/ACA ribonucleoprotein complex subunit DKC1 OS=Homo sapiens OX=9606 GN=DKC1 PE=1 SV=3
91	1	swissprot_human	sp|Q9Y5A9|YTHD2_HUMAN	48	62467	7	2	6	2	0.15	sp|Q9Y5A9|YTHD2_HUMAN YTH domain-containing family protein 2 OS=Homo sapiens OX=9606 GN=YTHDF2 PE=1 SV=2
92	1	swissprot_human	sp|P62269|RS18_HUMAN	47	17708	1	1	1	1	0.27	sp|P62269|RS18_HUMAN 40S ribosomal protein S18 OS=Homo sapiens OX=9606 GN=RPS18 PE=1 SV=3
93	1	swissprot_human	sp|P81605|DCD_HUMAN	46	11391	3	3	2	2	1.06	sp|P81605|DCD_HUMAN Dermcidin OS=Homo sapiens OX=9606 GN=DCD PE=1 SV=2
94	1	swissprot_human		46	73672	5	2	5	2	0.12	sp|P31040|SDHA_HUMAN Succinate

			sp|P31040|SDHA_HUMAN								dehydrogenase [ubiquinone] flavoprotein subunit, mitochondrial OS=Homo sapiens OX=9606 GN=SDHA PE=1 SV=2
95	1	swissprot_human	sp|Q5VTE0|EF1A3_HUMAN	45	50495	4	2	4	2	0.18	sp|Q5VTE0|EF1A3_HUMAN Putative elongation factor 1-alpha-like 3 OS=Homo sapiens OX=9606 GN=EEF1A1P5 PE=5 SV=1
96	1	swissprot_human	sp|Q9NUL3|STAU2_HUMAN	45	62797	5	2	4	2	0.14	sp|Q9NUL3|STAU2_HUMAN Double-stranded RNA-binding protein Staufen homolog 2 OS=Homo sapiens OX=9606 GN=STAU2 PE=1 SV=2
97	1	swissprot_human	sp|Q8NHQ9|DDX55_HUMAN	45	69073	8	1	8	1	0.06	sp|Q8NHQ9|DDX55_HUMAN ATP-dependent RNA helicase DDX55 OS=Homo sapiens OX=9606 GN=DDX55 PE=1 SV=3

98	1	swissprot_human	sp|Q86XN8|MEX3D_HUMAN	45	65641	3	1	3	1	0.07	sp|Q86XN8|MEX3D_HUMAN RNA-binding protein MEX3D OS=Homo sapiens OX=9606 GN=MEX3D PE=1 SV=3
99	1	swissprot_human	sp|Q7Z2W4|ZCCHV_HUMAN	44	103135	3	2	3	2	0.09	sp|Q7Z2W4|ZCCHV_HUMAN Zinc finger CCCH-type antiviral protein 1 OS=Homo sapiens OX=9606 GN=ZC3HAV1 PE=1 SV=3
100	1	swissprot_human	sp|Q5D862|FILA2_HUMAN	42	249296	3	1	3	1	0.02	sp|Q5D862|FILA2_HUMAN Filaggrin-2 OS=Homo sapiens OX=9606 GN=FLG2 PE=1 SV=1
52	2	swissprot_human	sp|Q92945|FUBP2_HUMAN	41	73355	3	2	3	2	0.12	sp|Q92945|FUBP2_HUMAN Far upstream element-binding protein 2 OS=Homo sapiens OX=9606 GN=KHSRP PE=1 SV=4
101	1	swissprot_human	sp|Q9Y2H1|ST38L_HUMAN	41	54196	8	1	7	1	0.08	sp|Q9Y2H1|ST38L_HUMAN Serine/threonine-protein kinase 38-like OS=Homo sapiens

											OX=9606 GN=STK38L PE=1 SV=3
102	1	swissprot_human	sp|P23588|IF4B_HUMAN	41	69167	7	3	5	3	0.2	sp|P23588|IF4B_HUMAN Eukaryotic translation initiation factor 4B OS=Homo sapiens OX=9606 GN=EIF4B PE=1 SV=2
103	1	swissprot_human	sp|P62277|RS13_HUMAN	41	17212	5	3	3	3	1.07	sp|P62277|RS13_HUMAN 40S ribosomal protein S13 OS=Homo sapiens OX=9606 GN=RPS13 PE=1 SV=2
104	1	swissprot_human	sp|Q9NUQ6|SPS2L_HUMAN	41	62204	5	2	5	2	0.15	sp|Q9NUQ6|SPS2L_HUMAN SPATS2-like protein OS=Homo sapiens OX=9606 GN=SPATS2L PE=1 SV=2
105	1	swissprot_human	sp|Q9H845|ACAD9_HUMAN	41	69344	7	3	7	3	0.2	sp|Q9H845|ACAD9_HUMAN Complex I assembly factor ACAD9, mitochondrial OS=Homo sapiens OX=9606 GN=ACAD9 PE=1 SV=1
106	1	swissprot_human		41	83349	9	3	8	3	0.17	sp|Q92499|DDX1_HUMAN ATP-dependent RNA

			sp|Q92499|DDX1_HUMAN								helicase DDX1 OS=Homo sapiens OX=9606 GN=DDX1 PE=1 SV=2
107	1	swissprot_human	sp|P02545|LMNA_HUMAN	41	74380	8	2	8	2	0.12	sp|P02545|LMNA_HUMAN Prelamin-A/C OS=Homo sapiens OX=9606 GN=LMNA PE=1 SV=1
108	1	swissprot_human	sp|P68871|HBB_HUMAN	40	16102	2	2	2	2	0.68	sp|P68871|HBB_HUMAN Hemoglobin subunit beta OS=Homo sapiens OX=9606 GN=HBB PE=1 SV=2
109	1	swissprot_human	sp|P17812|PYRG1_HUMAN	40	67332	5	1	5	1	0.07	sp|P17812|PYRG1_HUMAN CTP synthase 1 OS=Homo sapiens OX=9606 GN=CTPS1 PE=1 SV=2
91	2	swissprot_human	sp|Q7Z739|YTHD3_HUMAN	39	63936	4	2	4	2	0.14	sp|Q7Z739|YTHD3_HUMAN YTH domain-containing family protein 3 OS=Homo sapiens OX=9606 GN=YTHDF3 PE=1 SV=1

110	1	swissprot_human	sp|Q8N5A5|ZGPAT_HUMAN	39	57780	3	2	3	2	0.16	sp|Q8N5A5|ZGPAT_HUMAN Zinc finger CCCH-type with G patch domain-containing protein OS=Homo sapiens OX=9606 GN=ZGPAT PE=1 SV=3
111	1	swissprot_human	sp|P15924|DESP_HUMAN	39	334021	33	3	24	3	0.04	sp|P15924|DESP_HUMAN Desmoplakin OS=Homo sapiens OX=9606 GN=DSP PE=1 SV=3
112	1	swissprot_human	sp|O00257|CBX4_HUMAN	39	61672	4	3	4	3	0.23	sp|O00257|CBX4_HUMAN E3 SUMO-protein ligase CBX4 OS=Homo sapiens OX=9606 GN=CBX4 PE=1 SV=3
113	1	swissprot_human	sp|Q9Y262|EIF3L_HUMAN	39	66912	4	1	4	1	0.07	sp|Q9Y262|EIF3L_HUMAN Eukaryotic translation initiation factor 3 subunit L OS=Homo sapiens OX=9606 GN=EIF3L PE=1 SV=1
114	1	swissprot_human	sp|Q9H6R0|DHX33_HUMAN	38	79565	3	1	3	1	0.05	sp|Q9H6R0|DHX33_HUMAN ATP-dependent RNA helicase DHX33

											OS=Homo sapiens OX=9606 GN=DHX33 PE=1 SV=2
115	1	swissprot_human	sp|Q02413|DSG1_HUMAN	38	114702	3	1	3	1	0.04	sp|Q02413|DSG1_HUMAN Desmoglein-1 OS=Homo sapiens OX=9606 GN=DSG1 PE=1 SV=2
116	1	swissprot_human	sp|Q9Y3Z3|SAMH1_HUMAN	37	72896	5	1	5	1	0.06	sp|Q9Y3Z3|SAMH1_HUMAN Deoxynucleoside triphosphate triphosphohydrolase SAMHD1 OS=Homo sapiens OX=9606 GN=SAMHD1 PE=1 SV=2
117	1	swissprot_human	sp|Q9NVP1|DDX18_HUMAN	37	75702	2	2	2	2	0.12	sp|Q9NVP1|DDX18_HUMAN ATP-dependent RNA helicase DDX18 OS=Homo sapiens OX=9606 GN=DDX18 PE=1 SV=2
118	1	swissprot_human	sp|Q9P219|DAPLE_HUMAN	37	229231	12	1	9	1	0.02	sp|Q9P219|DAPLE_HUMAN Protein Daple OS=Homo sapiens OX=9606

											GN=CCDC88C PE=1 SV=3
119	1	swissprot_human	sp|P28288|ABCD3_HUMAN	37	75941	7	2	6	2	0.12	sp|P28288|ABCD3_HUMAN ATP-binding cassette sub-family D member 3 OS=Homo sapiens OX=9606 GN=ABCD3 PE=1 SV=1
120	1	swissprot_human	sp|Q15233|NONO_HUMAN	37	54311	7	3	7	3	0.26	sp|Q15233|NONO_HUMAN Non-POU domain-containing octamer-binding protein OS=Homo sapiens OX=9606 GN=NONO PE=1 SV=4
121	1	swissprot_human	sp|Q9H4L4|SENP3_HUMAN	37	65596	3	2	3	2	0.14	sp|Q9H4L4|SENP3_HUMAN Sentrin-specific protease 3 OS=Homo sapiens OX=9606 GN=SENP3 PE=1 SV=2
122	1	swissprot_human	sp|P04040|CATA_HUMAN	37	59947	2	1	2	1	0.07	sp|P04040|CATA_HUMAN Catalase OS=Homo sapiens OX=9606 GN=CAT PE=1 SV=3
123	1	swissprot_human		37	60054	6	1	5	1	0.07	sp|Q9Y2X3|NOP58_HUMAN Nucleolar protein 58

			sp|Q9Y2X3|NOP58_HUMAN								OS=Homo sapiens OX=9606 GN=NOP58 PE=1 SV=1
124	1	swissprot_human	sp|Q15428|SF3A2_HUMAN	36	49338	3	1	2	1	0.09	sp|Q15428|SF3A2_HUMAN Splicing factor 3A subunit 2 OS=Homo sapiens OX=9606 GN=SF3A2 PE=1 SV=2
125	1	swissprot_human	sp|Q8WUA2|PPIL4_HUMAN	36	57703	4	2	4	2	0.16	sp|Q8WUA2|PPIL4_HUMAN Peptidyl-prolyl cis-trans isomerase-like 4 OS=Homo sapiens OX=9606 GN=PPIL4 PE=1 SV=1
126	1	swissprot_human	sp|P23246|SFPQ_HUMAN	36	76216	7	4	6	3	0.18	sp|P23246|SFPQ_HUMAN Splicing factor, proline- and glutamine-rich OS=Homo sapiens OX=9606 GN=SFPQ PE=1 SV=2
127	1	swissprot_human	sp|Q8WU90|ZC3HF_HUMAN	36	48972	6	1	6	1	0.09	sp|Q8WU90|ZC3HF_HUMAN Zinc finger CCCH domain-containing protein 15 OS=Homo sapiens OX=9606 GN=ZC3H15 PE=1 SV=1

128	1	swissprot_human	sp|P39023|RL3_HUMAN	36	46365	2	1	2	1	0.1	sp|P39023|RL3_HUMAN 60S ribosomal protein L3 OS=Homo sapiens OX=9606 GN=RPL3 PE=1 SV=2
129	1	swissprot_human	sp|O43663|PRC1_HUMAN	36	72246	4	1	4	1	0.06	sp|O43663|PRC1_HUMAN Protein regulator of cytokinesis 1 OS=Homo sapiens OX=9606 GN=PRC1 PE=1 SV=2
130	1	swissprot_human	sp|Q14684|RRP1B_HUMAN	36	84774	8	2	7	2	0.11	sp|Q14684|RRP1B_HUMAN Ribosomal RNA processing protein 1 homolog B OS=Homo sapiens OX=9606 GN=RRP1B PE=1 SV=3
131	1	swissprot_human	sp|Q96EY7|PTCD3_HUMAN	36	79184	3	1	3	1	0.06	sp|Q96EY7|PTCD3_HUMAN Pentatricopeptide repeat domain-containing protein 3, mitochondrial OS=Homo sapiens OX=9606 GN=PTCD3 PE=1 SV=3
132	1	swissprot_human	sp|Q14157|UBP2L_HUMAN	35	114579	5	2	4	1	0.04	sp|Q14157|UBP2L_HUMAN Ubiquitin-associated protein 2-like OS=Homo

											sapiens OX=9606 GN=UBAP2L PE=1 SV=2
133	1	swissprot_human	sp|P61626|LYSC_HUMAN	35	16982	3	1	3	1	0.28	sp|P61626|LYSC_HUMAN Lysozyme C OS=Homo sapiens OX=9606 GN=LYZ PE=1 SV=1
134	1	swissprot_human	sp|P78347|GTF2I_HUMAN	35	112859	10	3	9	3	0.12	sp|P78347|GTF2I_HUMAN General transcription factor II-I OS=Homo sapiens OX=9606 GN=GTF2I PE=1 SV=2
135	1	swissprot_human	sp|Q49A26|GLYR1_HUMAN	34	61079	2	1	2	1	0.07	sp|Q49A26|GLYR1_HUMAN Putative oxidoreductase GLYR1 OS=Homo sapiens OX=9606 GN=GLYR1 PE=1 SV=4
136	1	swissprot_human	sp|O15371|EIF3D_HUMAN	34	64560	4	2	4	2	0.14	sp|O15371|EIF3D_HUMAN Eukaryotic translation initiation factor 3 subunit D OS=Homo sapiens OX=9606 GN=EIF3D PE=1 SV=1
137	1	swissprot_human	sp|Q9P0U3|SENP1_HUMAN	34	74005	2	1	2	1	0.06	sp|Q9P0U3|SENP1_HUMAN Sentrin-specific protease 1 OS=Homo

											sapiens OX=9606 GN=SENP1 PE=1 SV=2
138	1	swissprot_human	sp|Q96SZ6|CK5P1_HUMAN	34	68501	3	1	3	1	0.06	sp|Q96SZ6|CK5P1_HUMAN CDK5 regulatory subunit-associated protein 1 OS=Homo sapiens OX=9606 GN=CDK5RAP1 PE=1 SV=2
139	1	swissprot_human	sp|P62750|RL23A_HUMAN	34	17684	1	1	1	1	0.27	sp|P62750|RL23A_HUMAN 60S ribosomal protein L23a OS=Homo sapiens OX=9606 GN=RPL23A PE=1 SV=1
140	1	swissprot_human	sp|O00567|NOP56_HUMAN	34	66408	8	3	7	3	0.21	sp|O00567|NOP56_HUMAN Nucleolar protein 56 OS=Homo sapiens OX=9606 GN=NOP56 PE=1 SV=4
141	1	swissprot_human	sp|Q9P2N5|RBM27_HUMAN	34	119101	2	2	2	2	0.07	sp|Q9P2N5|RBM27_HUMAN RNA-binding protein 27 OS=Homo sapiens OX=9606 GN=RBM27 PE=1 SV=2
142	1	swissprot_human		33	60549	4	2	4	2	0.15	sp|O60678|ANM3_HUMAN Protein arginine N-

			sp|O60678|ANM3_HUMAN								methyltransferase 3 OS=Homo sapiens OX=9606 GN=PRMT3 PE=1 SV=4
143	1	swissprot_human	sp|P02768|ALBU_HUMAN	33	71317	3	1	2	1	0.06	sp|P02768|ALBU_HUMAN Serum albumin OS=Homo sapiens OX=9606 GN=ALB PE=1 SV=2
144	1	swissprot_human	sp|Q9HCD5|NCOA5_HUMAN	32	65725	2	1	2	1	0.07	sp|Q9HCD5|NCOA5_HUMAN Nuclear receptor coactivator 5 OS=Homo sapiens OX=9606 GN=NCOA5 PE=1 SV=2
145	1	swissprot_human	sp|P48200|IREB2_HUMAN	32	106018	3	1	3	1	0.04	sp|P48200|IREB2_HUMAN Iron-responsive element-binding protein 2 OS=Homo sapiens OX=9606 GN=IREB2 PE=1 SV=4
146	1	swissprot_human	sp|P49321|NASP_HUMAN	31	85471	1	1	1	1	0.05	sp|P49321|NASP_HUMAN Nuclear autoantigenic sperm protein OS=Homo sapiens OX=9606 GN=NASP PE=1 SV=2

147	1	swissprot_human	sp|Q02086|SP2_HUMAN	31	65487	1	1	1	1	0.07	sp|Q02086|SP2_HUMAN Transcription factor Sp2 OS=Homo sapiens OX=9606 GN=SP2 PE=1 SV=3
148	1	swissprot_human	sp|Q8TAT5|NEIL3_HUMAN	30	69265	2	1	2	1	0.06	sp|Q8TAT5|NEIL3_HUMAN Endonuclease 8-like 3 OS=Homo sapiens OX=9606 GN=NEIL3 PE=1 SV=3
149	1	swissprot_human	sp|Q96ME7|ZN512_HUMAN	30	65724	3	1	3	1	0.07	sp|Q96ME7|ZN512_HUMAN Zinc finger protein 512 OS=Homo sapiens OX=9606 GN=ZNF512 PE=1 SV=2
150	1	swissprot_human	sp|Q9Y6J9|TAF6L_HUMAN	29	68342	4	2	4	2	0.13	sp|Q9Y6J9|TAF6L_HUMAN TAF6-like RNA polymerase II p300/CBP-associated factor-associated factor 65 kDa subunit 6L OS=Homo sapiens OX=9606 GN=TAF6L PE=1 SV=1
151	1	swissprot_human	sp|Q9UJS0|CMC2_HUMAN	29	74528	4	1	4	1	0.06	sp|Q9UJS0|CMC2_HUMAN Calcium-binding mitochondrial carrier

											protein Aralar2 OS=Homo sapiens OX=9606 GN=SLC25A13 PE=1 SV=2
152	1	swissprot_human	sp|P40939|ECHA_HUMAN	29	83688	5	3	5	3	0.16	sp|P40939|ECHA_HUMAN Trifunctional enzyme subunit alpha, mitochondrial OS=Homo sapiens OX=9606 GN=HADHA PE=1 SV=2
153	1	swissprot_human	sp|O94776|MTA2_HUMAN	29	75717	2	1	2	1	0.06	sp|O94776|MTA2_HUMAN Metastasis-associated protein MTA2 OS=Homo sapiens OX=9606 GN=MTA2 PE=1 SV=1
154	1	swissprot_human	sp|Q9UKF6|CPSF3_HUMAN	28	78120	2	1	2	1	0.06	sp|Q9UKF6|CPSF3_HUMAN Cleavage and polyadenylation specificity factor subunit 3 OS=Homo sapiens OX=9606 GN=CPSF3 PE=1 SV=1
155	1	swissprot_human	sp|P57772|SELB_HUMAN	28	65890	4	2	4	2	0.14	sp|P57772|SELB_HUMAN Selenocysteine-specific elongation factor

											OS=Homo sapiens OX=9606 GN=EEFSEC PE=1 SV=4
156	1	swissprot_human	sp|Q1A5X6|IQCJ_HUMAN	28	18215	1	1	1	1	0.26	sp|Q1A5X6|IQCJ_HUMAN IQ domain-containing protein J OS=Homo sapiens OX=9606 GN=IQCJ PE=2 SV=1
96	2	swissprot_human	sp|O95793|STAU1_HUMAN	27	63428	5	2	4	2	0.14	sp|O95793|STAU1_HUMAN Double-stranded RNA-binding protein Staufen homolog 1 OS=Homo sapiens OX=9606 GN=STAU1 PE=1 SV=2
157	1	swissprot_human	sp|P16989|YBOX3_HUMAN	27	40066	1	1	1	1	0.11	sp|P16989|YBOX3_HUMAN Y-box-binding protein 3 OS=Homo sapiens OX=9606 GN=YBX3 PE=1 SV=4
158	1	swissprot_human	sp|P01040|CYTA_HUMAN	27	11000	1	1	1	1	0.45	sp|P01040|CYTA_HUMAN Cystatin-A OS=Homo sapiens OX=9606 GN=CSTA PE=1 SV=1
159	1	swissprot_human		27	74037	1	1	1	1		sp|Q96QH2|PRAM_HUMAN PML-RARA-

			sp|Q96QH2|PRAM_HUMAN								regulated adapter molecule 1 OS=Homo sapiens OX=9606 GN=PRAM1 PE=1 SV=3
160	1	swissprot_human	sp|Q1ED39|KNOP1_HUMAN	26	51728	2	1	2	1	0.09	sp|Q1ED39|KNOP1_HUMAN Lysine-rich nucleolar protein 1 OS=Homo sapiens OX=9606 GN=KNOP1 PE=1 SV=1
161	1	swissprot_human	sp|P12956|XRCC6_HUMAN	26	70084	9	4	9	4	0.28	sp|P12956|XRCC6_HUMAN X-ray repair cross-complementing protein 6 OS=Homo sapiens OX=9606 GN=XRCC6 PE=1 SV=2
162	1	swissprot_human	sp|O43242|PSMD3_HUMAN	26	61054	4	2	4	2	0.15	sp|O43242|PSMD3_HUMAN 26S proteasome non-ATPase regulatory subunit 3 OS=Homo sapiens OX=9606 GN=PSMD3 PE=1 SV=2
163	1	swissprot_human	sp|Q9BTX1|NDC1_HUMAN	26	76940	2	1	2	1	0.06	sp|Q9BTX1|NDC1_HUMAN Nucleoporin NDC1 OS=Homo sapiens

											OX=9606 GN=NDC1 PE=1 SV=2
164	1	swissprot_human	sp|P14923|PLAK_HUMAN	26	82434	5	2	4	1	0.05	sp|P14923|PLAK_HUMAN Junction plakoglobin OS=Homo sapiens OX=9606 GN=JUP PE=1 SV=3
165	1	swissprot_human	sp|Q96MX3|ZNF48_HUMAN	26	69203	4	2	4	2	0.13	sp|Q96MX3|ZNF48_HUMAN Zinc finger protein 48 OS=Homo sapiens OX=9606 GN=ZNF48 PE=1 SV=2
166	1	swissprot_human	sp|Q8NBJ4|GOLM1_HUMAN	26	45477	2	1	2	1	0.1	sp|Q8NBJ4|GOLM1_HUMAN Golgi membrane protein 1 OS=Homo sapiens OX=9606 GN=GOLM1 PE=1 SV=1
167	1	swissprot_human	sp|Q96KC8|DNJC1_HUMAN	26	64185	3	1	3	1	0.07	sp|Q96KC8|DNJC1_HUMAN DnaJ homolog subfamily C member 1 OS=Homo sapiens OX=9606 GN=DNAJC1 PE=1 SV=1
168	1	swissprot_human	sp|P62979|RS27A_HUMAN	26	18296	3	2	2	2	0.58	sp|P62979|RS27A_HUMAN Ubiquitin-40S ribosomal protein S27a

											OS=Homo sapiens OX=9606 GN=RPS27A PE=1 SV=2
169	1	swissprot_human	sp|Q9NWK9|BCD1_HUMAN	26	54511	3	1	3	1	0.08	sp|Q9NWK9|BCD1_HUMAN Box C/D snoRNA protein 1 OS=Homo sapiens OX=9606 GN=ZNHIT6 PE=1 SV=1
170	1	swissprot_human	sp|P62851|RS25_HUMAN	26	13791	3	2	3	2	0.83	sp|P62851|RS25_HUMAN 40S ribosomal protein S25 OS=Homo sapiens OX=9606 GN=RPS25 PE=1 SV=1
171	1	swissprot_human	sp|Q6P1J9|CDC73_HUMAN	26	60653	4	2	3	2	0.15	sp|Q6P1J9|CDC73_HUMAN Parafibromin OS=Homo sapiens OX=9606 GN=CDC73 PE=1 SV=1
172	1	swissprot_human	sp|Q08554|DSC1_HUMAN	25	101406	2	1	2	1	0.04	sp|Q08554|DSC1_HUMAN Desmocollin-1 OS=Homo sapiens OX=9606 GN=DSC1 PE=1 SV=2
173	1	swissprot_human	sp|Q15046|SYK_HUMAN	25	68461	5	1	5	1	0.06	sp|Q15046|SYK_HUMAN Lysine--tRNA ligase OS=Homo sapiens

											OX=9606 GN=KARS1 PE=1 SV=3
174	1	swissprot_human	sp|P19525|E2AK2_HUMAN	25	62512	5	1	4	1	0.07	sp|P19525|E2AK2_HUMAN Interferon-induced, double-stranded RNA-activated protein kinase OS=Homo sapiens OX=9606 GN=EIF2AK2 PE=1 SV=2
175	1	swissprot_human	sp|Q0D2J5|ZN763_HUMAN	25	47438	3	1	2	1	0.09	sp|Q0D2J5|ZN763_HUMAN Zinc finger protein 763 OS=Homo sapiens OX=9606 GN=ZNF763 PE=2 SV=2
176	1	swissprot_human	sp|Q92522|H1X_HUMAN	25	22474	1	1	1	1	0.21	sp|Q92522|H1X_HUMAN Histone H1x OS=Homo sapiens OX=9606 GN=H1FX PE=1 SV=1
177	1	swissprot_human	sp|P62701|RS4X_HUMAN	25	29807	1	1	1	1		sp|P62701|RS4X_HUMAN 40S ribosomal protein S4, X isoform OS=Homo sapiens OX=9606 GN=RPS4X PE=1 SV=2
178	1	swissprot_human	sp|Q9NXV6|CARF_HUMAN	25	61544	3	1	3	1	0.07	sp|Q9NXV6|CARF_HUMAN CDKN2A-interacting protein OS=Homo

											sapiens OX=9606 GN=CDKN2AIP PE=1 SV=3
179	1	swissprot_human	sp|Q9BWE0|REPI1_HUMAN	25	65530	1	1	1	1	0.07	sp|Q9BWE0|REPI1_HUMAN Replication initiator 1 OS=Homo sapiens OX=9606 GN=REPIN1 PE=1 SV=1
180	1	swissprot_human	sp|Q9NRG9|AAAS_HUMAN	24	60392	2	1	2	1	0.07	sp|Q9NRG9|AAAS_HUMAN Aladin OS=Homo sapiens OX=9606 GN=AAAS PE=1 SV=1
181	1	swissprot_human	sp|Q9Y5Q8|TF3C5_HUMAN	24	59989	1	1	1	1	0.07	sp|Q9Y5Q8|TF3C5_HUMAN General transcription factor 3C polypeptide 5 OS=Homo sapiens OX=9606 GN=GTF3C5 PE=1 SV=2
182	1	swissprot_human	sp|P30419|NMT1_HUMAN	24	57112	4	2	3	2	0.16	sp|P30419|NMT1_HUMAN Glycylpeptide N-tetradecanoyltransferase 1 OS=Homo sapiens OX=9606 GN=NMT1 PE=1 SV=2

87	2	swissprot_human	sp|Q12906|ILF3_HUMAN	23	95678	7	3	6	3	0.14	sp|Q12906|ILF3_HUMAN Interleukin enhancer-binding factor 3 OS=Homo sapiens OX=9606 GN=ILF3 PE=1 SV=3
183	1	swissprot_human	sp|Q06265|EXOS9_HUMAN	23	49545	4	1	4	1	0.09	sp|Q06265|EXOS9_HUMAN Exosome complex component RRP45 OS=Homo sapiens OX=9606 GN=EXOSC9 PE=1 SV=3
184	1	swissprot_human	sp|Q53GS9|SNUT2_HUMAN	23	65739	2	1	2	1	0.07	sp|Q53GS9|SNUT2_HUMAN U4/U6.U5 tri-snRNP-associated protein 2 OS=Homo sapiens OX=9606 GN=USP39 PE=1 SV=2
185	1	swissprot_human	sp|Q9BQ39|DDX50_HUMAN	23	83084	4	1	4	1	0.05	sp|Q9BQ39|DDX50_HUMAN ATP-dependent RNA helicase DDX50 OS=Homo sapiens OX=9606 GN=DDX50 PE=1 SV=1
186	1	swissprot_human	sp|Q14739|LBR_HUMAN	23	71057	3	1	3	1	0.06	sp|Q14739|LBR_HUMAN Delta(14)-sterol

											reductase LBR OS=Homo sapiens OX=9606 GN=LBR PE=1 SV=2
187	1	swissprot_human	sp|Q4G0J3|LARP7_HUMAN	22	67143	3	1	3	1	0.07	sp|Q4G0J3|LARP7_HUMAN La-related protein 7 OS=Homo sapiens OX=9606 GN=LARP7 PE=1 SV=1
188	1	swissprot_human	sp|P25490|TYY1_HUMAN	21	45141	2	1	2	1	0.1	sp|P25490|TYY1_HUMAN Transcriptional repressor protein YY1 OS=Homo sapiens OX=9606 GN=YY1 PE=1 SV=2
189	1	swissprot_human	sp|Q9NQ55|SSF1_HUMAN	21	53446	3	1	3	1		sp|Q9NQ55|SSF1_HUMAN Suppressor of SWI4 1 homolog OS=Homo sapiens OX=9606 GN=PPAN PE=2 SV=1
190	1	swissprot_human	sp|P20930|FILA_HUMAN	21	435036	17	1	11	1	0.01	sp|P20930|FILA_HUMAN Filaggrin OS=Homo sapiens OX=9606 GN=FLG PE=1 SV=3
191	1	swissprot_human	sp|P62424|RL7A_HUMAN	21	30148	2	1	2	1	0.15	sp|P62424|RL7A_HUMAN 60S ribosomal protein

											L7a OS=Homo sapiens OX=9606 GN=RPL7A PE=1 SV=2
192	1	swissprot_human	sp|P13797|PLST_HUMAN	20	71279	3	1	2	1	0.06	sp|P13797|PLST_HUMAN Plastin-3 OS=Homo sapiens OX=9606 GN=PLS3 PE=1 SV=4
193	1	swissprot_human	sp|P02788|TRFL_HUMAN	20	80014	4	1	4	1	0.05	sp|P02788|TRFL_HUMAN Lactotransferrin OS=Homo sapiens OX=9606 GN=LTF PE=1 SV=6
194	1	swissprot_human	sp|P16104|H2AX_HUMAN	20	15135	2	1	2	1	0.32	sp|P16104|H2AX_HUMAN Histone H2AX OS=Homo sapiens OX=9606 GN=H2AFX PE=1 SV=2
195	1	swissprot_human	sp|Q14527|HLTF_HUMAN	20	114883	4	2	4	2	0.08	sp|Q14527|HLTF_HUMAN Helicase-like transcription factor OS=Homo sapiens OX=9606 GN=HLTF PE=1 SV=2
196	1	swissprot_human	sp|Q9UG63|ABCF2_HUMAN	20	71815	6	1	6	1	0.06	sp|Q9UG63|ABCF2_HUMAN ATP-binding cassette sub-family F

											member 2 OS=Homo sapiens OX=9606 GN=ABCF2 PE=1 SV=2
197	1	swissprot_human	sp|O15213|WDR46_HUMAN	20	68485	3	2	3	2	0.13	sp|O15213|WDR46_HUMAN WD repeat-containing protein 46 OS=Homo sapiens OX=9606 GN=WDR46 PE=1 SV=3
198	1	swissprot_human	sp|P09874|PARP1_HUMAN	20	113811	10	1	9	1	0.04	sp|P09874|PARP1_HUMAN Poly [ADP-ribose] polymerase 1 OS=Homo sapiens OX=9606 GN=PARP1 PE=1 SV=4
199	1	swissprot_human	sp|Q5QP82|DCA10_HUMAN	19	61627	1	1	1	1	0.07	sp|Q5QP82|DCA10_HUMAN DDB1- and CUL4-associated factor 10 OS=Homo sapiens OX=9606 GN=DCAF10 PE=1 SV=1
200	1	swissprot_human	sp|P47914|RL29_HUMAN	19	17798	3	1	2	1	0.27	sp|P47914|RL29_HUMAN 60S ribosomal protein L29 OS=Homo sapiens OX=9606 GN=RPL29 PE=1 SV=2

201	1	swissprot_h uman	sp|Q8NCQ2|CNAS1_HUMAN	19	1623 2	1	1	1	1	0.29	sp|Q8NCQ2|CNAS1_HU MAN Uncharacterized protein CSNK1G2-AS1 OS=Homo sapiens OX=9606 GN=CSNK1G2-AS1 PE=2 SV=2
202	1	swissprot_h uman	sp|Q16526|CRY1_HUMAN	19	6715 1	4	1	4	1	0.07	sp|Q16526|CRY1_HUM AN Cryptochrome-1 OS=Homo sapiens OX=9606 GN=CRY1 PE=1 SV=1
203	1	swissprot_h uman	sp|Q9H0W5|CCDC8_HUMAN	18	5945 3	3	1	2	1	0.07	sp|Q9H0W5|CCDC8_HU MAN Coiled-coil domain-containing protein 8 OS=Homo sapiens OX=9606 GN=CCDC8 PE=1 SV=2
204	1	swissprot_h uman	sp|Q9BUI4|RPC3_HUMAN	18	6097 2	3	1	3	1	0.07	sp|Q9BUI4|RPC3_HUM AN DNA-directed RNA polymerase III subunit RPC3 OS=Homo sapiens OX=9606 GN=POLR3C PE=1 SV=1
205	1	swissprot_h uman	sp|P06702|S10A9_HUMAN	18	1329 1	1	1	1	1	0.37	sp|P06702|S10A9_HUM AN Protein S100-A9

											OS=Homo sapiens OX=9606 GN=S100A9 PE=1 SV=1
206	1	swissprot_human	sp|Q86X55|CARM1_HUMAN	17	66382	3	1	3	1	0.07	sp|Q86X55|CARM1_HUMAN Histone-arginine methyltransferase CARM1 OS=Homo sapiens OX=9606 GN=CARM1 PE=1 SV=3
207	1	swissprot_human	sp|Q13547|HDAC1_HUMAN	17	55638	3	1	3	1	0.08	sp|Q13547|HDAC1_HUMAN Histone deacetylase 1 OS=Homo sapiens OX=9606 GN=HDAC1 PE=1 SV=1
208	1	swissprot_human	sp|Q14247|SRC8_HUMAN	17	61720	7	1	7	1	0.07	sp|Q14247|SRC8_HUMAN Src substrate cortactin OS=Homo sapiens OX=9606 GN=CTTN PE=1 SV=2
209	1	swissprot_human	sp|Q9Y618|NCOR2_HUMAN	17	274230	5	1	4	1	0.02	sp|Q9Y618|NCOR2_HUMAN Nuclear receptor corepressor 2 OS=Homo sapiens OX=9606 GN=NCOR2 PE=1 SV=3
210	1	swissprot_human		16	629213	8	1	8	1	0.01	sp|Q09666|AHNK_HUMAN Neuroblast

	1		sp|Q09666|AHNK_HUMAN								differentiation-associated protein AHNAK OS=Homo sapiens OX=9606 GN=AHNAK PE=1 SV=2
211	1	swissprot_human	sp|Q96L91|EP400_HUMAN	16	344702	1	1	1	1	0.01	sp|Q96L91|EP400_HUMAN E1A-binding protein p400 OS=Homo sapiens OX=9606 GN=EP400 PE=1 SV=4
212	1	swissprot_human	sp|O75127|PTCD1_HUMAN	16	79433	5	1	4	1	0.06	sp|O75127|PTCD1_HUMAN Pentatricopeptide repeat-containing protein 1, mitochondrial OS=Homo sapiens OX=9606 GN=PTCD1 PE=1 SV=2
213	1	swissprot_human	sp|P69905|HBA_HUMAN	16	15305	2	1	2	1	0.31	sp|P69905|HBA_HUMAN Hemoglobin subunit alpha OS=Homo sapiens OX=9606 GN=HBA1 PE=1 SV=2
214	1	swissprot_human	sp|Q15751|HERC1_HUMAN	16	538790	5	1	5	1	0.01	sp|Q15751|HERC1_HUMAN Probable E3 ubiquitin-protein ligase HERC1 OS=Homo

											sapiens OX=9606 GN=HERC1 PE=1 SV=2
215	1	swissprot_human	sp|Q8N999|CL029_HUMAN	15	38093	8	1	2	1	0.12	sp|Q8N999|CL029_HUMAN Uncharacterized protein C12orf29 OS=Homo sapiens OX=9606 GN=C12orf29 PE=1 SV=2
216	1	swissprot_human	sp|P62861|RS30_HUMAN	15	6644	1	1	1	1	0.83	sp|P62861|RS30_HUMAN 40S ribosomal protein S30 OS=Homo sapiens OX=9606 GN=FAU PE=1 SV=1
217	1	swissprot_human	sp|Q8TDD1|DDX54_HUMAN	15	98819	5	1	4	1	0.04	sp|Q8TDD1|DDX54_HUMAN ATP-dependent RNA helicase DDX54 OS=Homo sapiens OX=9606 GN=DDX54 PE=1 SV=2
218	1	swissprot_human	sp|Q9H0B6|KLC2_HUMAN	15	69291	3	1	2	1	0.06	sp|Q9H0B6|KLC2_HUMAN Kinesin light chain 2 OS=Homo sapiens OX=9606 GN=KLC2 PE=1 SV=1
219	1	swissprot_human	sp|P62263|RS14_HUMAN	14	16434	3	2	2	1	0.29	sp|P62263|RS14_HUMAN 40S ribosomal protein

											S14 OS=Homo sapiens OX=9606 GN=RPS14 PE=1 SV=3
220	1	swissprot_human	sp|Q6NW34|NEPRO_HUMAN	14	65366	3	1	3	1	0.07	sp|Q6NW34|NEPRO_HUMAN Nucleolus and neural progenitor protein OS=Homo sapiens OX=9606 GN=NEPRO PE=1 SV=3
221	1	swissprot_human	sp|Q8WVV9|HNRLL_HUMAN	14	60900	4	1	4	1	0.07	sp|Q8WVV9|HNRLL_HUMAN Heterogeneous nuclear ribonucleoprotein L-like OS=Homo sapiens OX=9606 GN=HNRNPLL PE=1 SV=1
222	1	swissprot_human	sp|Q7Z628|ARHG8_HUMAN	14	68154	1	1	1	1	0.06	sp|Q7Z628|ARHG8_HUMAN Neuroepithelial cell-transforming gene 1 protein OS=Homo sapiens OX=9606 GN=NET1 PE=1 SV=1
223	1	swissprot_human	sp|Q9NZ56|FMN2_HUMAN	13	181191	2	1	2	1	0.02	sp|Q9NZ56|FMN2_HUMAN Formin-2 OS=Homo sapiens OX=9606 GN=FMN2 PE=1 SV=4

224	1	swissprot_human	sp|Q5SYE7|NHSL1_HUMAN	13	172103	9	1	7	1	0.03	sp|Q5SYE7|NHSL1_HUMAN NHS-like protein 1 OS=Homo sapiens OX=9606 GN=NHSL1 PE=1 SV=2
225	1	swissprot_human	sp|P0DN79|CBSL_HUMAN	13	61175	2	1	2	1	0.07	sp|P0DN79|CBSL_HUMAN Cystathionine beta-synthase-like protein OS=Homo sapiens OX=9606 GN=CBSL PE=1 SV=1
226	1	swissprot_human	sp|P51911|CNN1_HUMAN	13	33321	3	1	1	1	0.14	sp|P51911|CNN1_HUMAN Calponin-1 OS=Homo sapiens OX=9606 GN=CNN1 PE=1 SV=2
